# Efficacy and safety of Chinese botanical drug decoctions for migraine: a Bayesian network meta-analysis

**DOI:** 10.3389/fphar.2026.1799920

**Published:** 2026-05-01

**Authors:** Fan Zhang, Haogeng Wang, Dianhui Yang

**Affiliations:** 1 College of Acupuncture-moxibustion and Tuina, Shandong University of Traditional Chinese Medicine, Jinan, Shandong, China; 2 Department of Acupuncture-moxibustion, The Affiliated Hospital of Shandong University of Traditional Chinese Medicine, Jinan, Shandong, China

**Keywords:** Chinese herbal decoction, efficacy and safety, migraine, network meta-analysis, systematic review

## Abstract

**Background:**

Migraine is a common neurological disorder that substantially affects patients’ quality of life. Chinese botanical drug decoctions are used in migraine management; however, the comparative efficacy and safety of different decoctions remain unclear. Therefore, this study aimed to compare multiple Chinese botanical drug decoctions for migraine using a Bayesian network meta-analysis.

**Methods:**

Seven electronic databases (PubMed, Embase, Cochrane Library, Web of Science, CNKI, Wanfang, and VIP) were systematically searched from inception to 20 October 2025. Randomized controlled trials evaluating Chinese botanical drug decoctions for migraine were included. Risk of bias was assessed using the ROB 2.0 tool. A Bayesian network meta-analysis was conducted using R software with the gemtc package. Four Markov chains were run simultaneously, with 50,000 iterations and a burn-in period of 20,000 iterations to ensure model convergence. Treatment ranking probabilities were estimated using the surface under the cumulative ranking curve (SUCRA).

**Results:**

A total of 57 randomized controlled trials involving 6,005 patients were included. Network meta-analysis suggested that Sanpian Decoction (SPT) was associated with greater reductions in visual Analog Scale (VAS) scores, while Xuefu Zhuyu Decoction (XFZYT) ranked highest for reducing headache frequency and duration. Tongqiao Huoxue Decoction (TQHXT) had the highest probability of being among the most effective interventions based on SUCRA rankings.

**Conclusion:**

Chinese botanical drug decoctions may be associated with improvements in migraine-related outcomes. Among the evaluated interventions, SPT may be associated with greater reductions in pain intensity, XFZYT may be associated with reductions in attack frequency and duration, and TQHXT had the highest SUCRA-based ranking for overall efficacy. However, these findings should be interpreted with caution due to variability in study quality and the indirect nature of comparisons.

**Systematic Review Registration:**

https://www.crd.york.ac.uk/PROSPERO/view/CRD420251176748.

## Background

Migraine is a common primary neurological disorder characterized by recurrent episodes of headache, often accompanied by nausea, vomiting, photophobia, and phonophobia (Chiang et al., 2024; Christensen and Levy, 2024). Some patients may experience visual, sensory, or language disturbances as prodromal symptoms before or during an attack (Ornello et al., 2024). Epidemiological studies indicate migraine exhibits a high global prevalence with distinct gender disparities and age distribution patterns, predominantly affecting young to middle-aged women (Eller et al., 2025). Due to its frequent attacks, prolonged course, and high recurrence rate, migraine significantly diminishes patients’ quality of life and work efficiency while imposing sustained impacts on healthcare resources and economic burdens. It has been classified by the World Health Organization as one of the leading causes of disability worldwide (Fernandes et al., 2024; Oliveira et al., 2024).

The exact pathogenesis of migraine remains incompletely understood, though it is widely recognized to involve multiple factors including neurovascular dysfunction, central nervous system sensitization, release of inflammatory mediators, and genetic susceptibility (Wang, 2025). Treatment approaches in modern medicine primarily focus on symptomatic relief and preventive therapy. Common acute-phase medications include nonsteroidal anti-inflammatory drugs (NSAIDs), triptans, and ergot derivatives. Preventive treatments predominantly utilize beta-blockers, antiepileptic drugs, antidepressants, and more recently, calcitonin gene-related peptide (CGRP) targeted medications (Ashraf and Goadsby, 2025; Mistry et al., 2024). While these medications can alleviate migraine symptoms or reduce attack frequency to some extent, significant challenges persist marked individual variations in efficacy, poor long-term treatment adherence, high rates of adverse reactions, and substantial financial burdens (Olofsson, 2024; Zhu XK. et al., 2025). Particularly concerning are the gastrointestinal reactions, cardiovascular adverse events, or central nervous system discomfort experienced by some patients after prolonged use of standard biomedical treatment, limiting their clinical application.

Given the limitations associated with currently available pharmacological treatments, there is growing interest in complementary and alternative approaches, including traditional medicine systems. Among these, Chinese botanical drug decoctions have been investigated as potential therapeutic options for migraine management. However, their comparative efficacy and safety remain to be systematically evaluated (Zhu Q. et al., 2025). Chinese botanical drug decoctions typically classifies migraines under categories such as “headache” or “hemicrania,” attributing their occurrence to the invasion of external pathogens like wind, cold, dampness, heat, and blood stasis, as well as imbalances in liver, spleen, and kidney functions, and disharmony between qi and blood (Huang et al., 2020). Chinese botanical drug decoctions, as a key therapeutic modality in Chinese botanical drug decoctions for migraine, emphasize pattern differentiation and holistic regulation (Chen et al., 2024). Through multi-targeted, multi-pathway synergistic effects, they aim to alleviate symptoms, reduce recurrence, and improve overall health status (Fan et al., 2024).

In recent years, with the advancement of evidence-based medicine, the number of randomized controlled trials (RCTs) evaluating botanical drug decoctions for migraine has increased. Findings suggest that various botanical drug decoctions demonstrate certain advantages in reducing migraine attack frequency, alleviating pain intensity, shortening attack duration, and improving associated symptoms, while exhibiting relatively good safety profiles (Lu et al., 2023). However, due to differences in study design, intervention protocols, outcome measures, and control measures, existing research conclusions remain inconsistent. Furthermore, most traditional meta-analyses are limited to pairwise comparisons, typically evaluating only the efficacy difference between a specific herbal decoction and a single control measure (Fan et al., 2024; Xiao et al., 2015). This approach struggles to simultaneously compare the relative efficacy and ranking of multiple botanical drug decoctions, thereby limiting its value in guiding clinical decision-making. Against this backdrop, this study aims to conduct a network meta-analysis of randomized controlled trials evaluating Chinese botanical drug decoctions for migraine treatment. This will be achieved through systematic retrieval of relevant domestic and international literature, application of rigorous methodological standards, and use of bias risk assessment tools. This study aims to provide evidence-based guidance for clinicians, helping to identify the effective Chinese botanical drug decoctions for migraine management and inform future research in this area.

## Methods

This study was conducted in accordance with the Preferred Reporting Items for Systematic Reviews and Meta-Analyses (PRISMA) guidelines (Page et al., 2021) and was registered in PROSPERO (CRD420251176748).

### Literature retrieval

Two researchers independently conducted literature searches, systematically retrieving studies from PubMed, Embase, Cochrane Library, Web of Science, China National Knowledge Infrastructure (CNKI), Wan fang Database, and VIP Database from each database’s inception to 20 October 2025. A combined strategy of subject headings and free-text terms was employed. Key search terms included “migraine,” “Chinese herbal decoction,” “traditional Chinese medicine,” and “herbal medicine”. To prevent omission of relevant studies, a retrospective search of references from included publications was also conducted. No language restrictions were applied during the search. The specific search strategy is detailed in Supplementary Table S1.

### Inclusion and exclusion criteria

#### Inclusion criteria

Population: The study subjects were patients with a confirmed diagnosis of migraine, meeting the diagnostic criteria of the International Classification of Headache Disorders (ICHD) or recognized clinical diagnostic standards. There were no restrictions based on gender, age, disease duration, or ethnicity.

Intervention: The experimental group received Chinese herbal decoction therapy (including Yangxue Pinggan Decoction (FYXPGT); Banxia Baizhu Tianma Decoction (BXBZTMT); Wuzhuyu Decoction (WZYT); Sanpian Decoction (SPT); Xuefu Zhuyu Decoction (XFZYT); Tongqiao Huoxue decoction (TQHXT); Mahuang Fuzi Xixin Decoction (MHFZXXT)), either as monotherapy or in combination with conventional treatment. These decoctions are widely used clinically to treat migraine and are clearly documented in the literature of traditional Chinese medicine for migraine. These decoctions have been evaluated in many clinical studies and described in detail in Chinese botanical drug decoctions classics. Therefore, they have high clinical application value and relevance and are suitable for the research object of this systematic review and network meta-analysis.

Comparator: The control group receives conventional standard biomedical treatment, placebo, or other Chinese botanical drug decoctions.

Outcome: Primary outcomes included visual Analog Scale (VAS); Headache Frequency; Headache Duration; Efficacy; Secondary outcomes were defined as adverse Events.

Study Design: This study included randomized controlled trials (RCTs).

#### Exclusion criteria

Non-randomized controlled studies, such as retrospective studies, case-control studies, cohort studies, case series, case reports, reviews, conference abstracts, or animal experiments.

Studies involving subjects with secondary headaches or those with concomitant severe neurological disorders, where migraine-specific data cannot be extracted independently.

Interventions involving non-decoction Chinese medicine therapies such as proprietary Chinese medicines, acupuncture, tuina massage, or acupoint plaster application; or combined Chinese botanical drug decoctions where the independent efficacy of decoction cannot be separated.

Studies where both treatment and control groups received identical Chinese medicine decoctions, differing only in dosage or duration.

Studies with unclear outcome measures or incomplete data that could not be obtained by contacting authors.

Duplicate publications: if the same study population appeared in multiple publications, only the one with the largest sample size or most complete information was included.

### Data extractions

Two authors independently screened the literature for inclusion by importing the literature into endnote according to the literature inclusion and exclusion criteria, the final included studies were used for data extraction using excel software and if there was a dispute about the literature screening then it would be discussed, or a third person would be sought to adjudicate. Data were managed using EndNote software for efficient screening. The extracted data contained basic characteristics of the study (first author, year of publication), basic characteristics of the population (sample size, gender, age), intervention, follow-up and outcome. To improve the transparency and reproducibility of herbal interventions, we systematically extracted and standardized the composition of each formula from the included studies and structured the information according to the ConPhyMP (Consensus on the Phytochemical Characterisation of Medicinal Plant Preparations) framework, ConPhyMP-checklists can be found in the Supplementary Material.

### Assessment of intervention reporting quality

In addition to extracting the names of the included decoctions, we assessed the adequacy of composition reporting for each intervention. For each formula, we distinguished between the official pharmacopoeial/regulatory or other authoritative reference composition, where identifiable, and the composition reported in the included study. Reporting quality was then classified as: 1 = adequate, when the intervention was described with sufficient detail to identify its constituent materials clearly; 2 = limited, when the composition was only partially specified or lacked adequate pharmacognostic/pharmaceutical characterization; and 3 = inadequate, when the intervention was insufficiently described to determine its composition reliably.

### Risk of bias

In the Meta-analysis of this study, we used the ROB 2.0 (Risk of Bias 2.0) tool (Lu et al., 2020) to assess the risk of bias of the included studies. Developed by the Cochrane Collaboration, the ROB 2.0 tool is a standardized tool for assessing the risk of bias in RCTs, which is aimed at systematically identifying and evaluating bias factors that may affect the validity of the results of the study. This improves the accuracy and reliability of Meta-analyses. The ROB 2.0 tool contains five key assessment domains: randomization process, intervention implementation, outcome measures, data reporting, and other sources of bias. Each domain is scored according to the transparency, reasonableness, and potential for bias in the study design and implementation, and is categorized as low risk, high risk, and uncertain risk. In conducting the assessment, two independent reviewers will score each domain based on the specifics of the study. Specifically, the randomization process assesses whether the study adopts an effective random assignment method to avoid selection bias; intervention implementation assesses whether there are deviations from the intended treatment plan that may affect treatment outcomes; outcome measures assess whether standardized, objective, and consistent measurement tools are used to avoid information bias; data reporting assesses whether there is selective reporting bias, which is the practice of reporting only favorable outcomes and ignoring outcomes that do not meet the expected outcomes; and other sources of bias focus on the impact of factors external to the study, such as funding sources and investigator conflicts of interest, on the study. Each area will be assessed based on the specific information in the study report, and if some information is missing or unclear, the reviewers will mark it as “uncertain” and try to resolve it by obtaining further information or communicating with the original authors. Ultimately, the risk of bias for a study will be assessed as low, high, or uncertain based on the results of the assessments in all areas.

### Grade assessment

To systematically assess the quality of the evidence included in the study, this research used the GRADING (Grading of Recommendations Assessment, Development, and Evaluation) (Guyatt et al., 2008) system to rate the final evidence. The GRADING system is a tool widely used in clinical research that comprehensively evaluates the quality of evidence by assessing factors such as study design, risk of bias, consistency, directness, and precision. According to the GRADING system, evidence quality is divided into four levels: high, moderate, low, and very low.

### Statistical analysis

Statistical analyses were performed using a Bayesian framework for network meta-analysis (Az et al., 2025) to compare the efficacy of different decoctions in treating migraine. First, a treatment network will be constructed by connecting studies that directly or indirectly compare two or more treatments. Each treatment will serve as a node, and edges between nodes represent direct comparisons between treatments performed in individual studies. Next, Bayesian network Meta-analysis was employed, treatment effects were expressed as mean differences (MD) for continuous outcomes and odds ratios (OR) for dichotomous outcomes with 95% credible intervals (CrI), a Bayesian approach allowing the introduction of prior distributions and the use of Markov chain Monte Carlo (MCMC) methods to generate posterior distributions of treatment effects. Four Markov chains were run simultaneously with different initial values. Each chain consisted of 50,000 iterations, with the first 20,000 iterations discarded as burn-in. Convergence was assessed using the Gelman–Rubin diagnostic and potential scale reduction factor (PSRF). In this study, Deviance Information Criterion (DIC) will be used to assess the transitivity assumption of the network Meta-analysis model. Specifically, two models will be constructed: one assuming consistency and the other assuming inconsistency. By comparing the DIC values of these two models, we can determine whether there is inconsistency in the network. To account for inter-study heterogeneity, a random effects model will be used for the analysis. Heterogeneity will be assessed by the I^2^ statistic, with significant heterogeneity indicated if the I^2^ value exceeds 50%. Network consistency will also be tested to ensure consistency between direct and indirect evidence. If inconsistency is found, possible causes will be explored, and the model will be adjusted through sensitivity analysis. All statistical analyses will be performed using R software (version 4.0.0) and the “gemtc” package, which is specifically designed for Bayesian network meta-analysis. A random effects model will be used in the analysis to account for potential heterogeneity between studies. This model allows for variability in treatment effects across studies and is more appropriate when there is diversity in study designs, populations, or interventions. Ranking of Treatments: Surface Under the Cumulative Ranking Curve (SUCRA) was used to rank the treatments based on their probability for each outcome.

## Results

### Literature retrieval results

**FIGURE 1:**
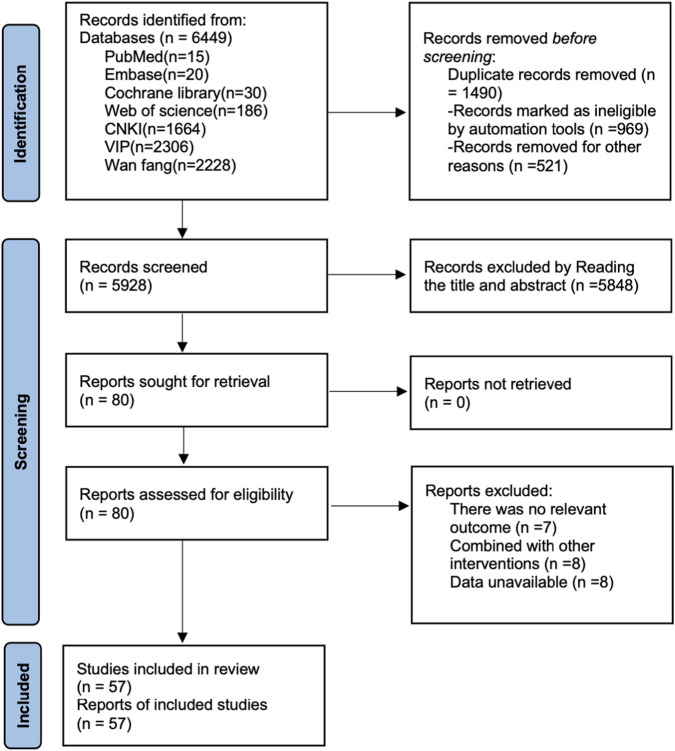
Literature search flow chart.

As shown in Figure 1, a total of 6,449 records was identified through database searches. After removing duplicates, 4,959 records remained. Following title and abstract screening, 101 articles underwent full-text review, and 57 randomized controlled trials were finally included.

### Basic characteristics of included studies

This study included a total of 57 articles involving 6,005 migraine patients. The overall gender ratio of participants was balanced, with an average age ranging from 26 to 65 years. The intervention and control groups demonstrated good comparability in baseline characteristics such as age and gender. Interventions involved various Chinese botanical drug decoctions, including YXPGT, BXBZTMT, WZYT, SPT, XFZYT, TQHXT, and MHFZXXT. All were administered orally, primarily twice daily, with treatment cycles typically lasting 2–4 weeks. A few studies extended treatment to 12 weeks or 3 months. Most studies did not explicitly report follow-up duration, with some ranging from 4 weeks to 1 year. Detailed baseline characteristics are presented in Supplementary Table S2.

### Reporting quality and reproducibility of the included decoctions

The reporting quality of the herbal interventions is summarized in Supplementary Table S3. Overall, the included studies showed substantial deficiencies in reporting the provenance, composition, and preparation of the investigated decoctions. Only a minority of studies provided sufficiently detailed information on both the core ingredients and administration procedures to support at least partial reproducibility, whereas many reports did not provide a complete ingredient list, did not identify an official pharmacopoeia or regulatory standard, and/or lacked sufficient preparation details. In several studies, the composition was reported incompletely or not at all, which precluded taxonomic validation of the relevant potential species and substantially limited reproducibility. Therefore, although the interventions were labelled under the same decoction names, the actual formulations may not have been standardized or fully comparable across studies.

### Risk of bias results

This study employed ROB2.0 for quality assessment. Results (Supplementary Figure S1; Supplementary Table S4) indicate that four studies failed to specify the randomization method used, leading to a rating of “Some concerns.” Most studies reported appropriate randomization; however, four studies did not clearly describe the randomization method and were rated as having “some concerns.” In addition, 16 studies did not clearly report blinding procedures, resulting in potential bias in intervention implementation. This study employed the GRADE approach, with results (Supplementary Table S5) indicating that evidence for the VAS; Headache Frequency; Headache Duration; Efficacy; and Adverse Events was classified as moderate.

### Results of consistency modeling

The current study used a random-effects model to compare the difference in DIC between consistent and inconsistent modeling, and the absolute value of the difference in DIC was <5, the results (Supplementary Table S6) indicate that Visual Analog Scale (VAS); Headache Frequency; Headache Duration; Efficacy; Adverse Events have consistency.

### Pairwise meta-analysis

This study employed a paired meta-analysis comparison. Results (Table 1) indicate that for the visual analogue scale, MHFZXXT vs. Control [MD = −1.52, 95%CrI (−2.81, −0.24)], SPT vs. Control [MD = −2.87, 95%CrI (−4.21, −1.73)], TQHXT vs. Control [MD = −1.37, 95%CrI (−2.56, −0.20)], and for headache frequency, SPT vs. Control [MD = −1.54, 95%CrI (−2.56, −0.53)], TQHXT vs. Control [MD = −1.63, 95%CrI (−2.37, −0.91)]. XFZYT vs. Control [MD = −1.70, 95%CrI (−2.28, −1.12)], For headache duration, Control vs. BXBZTMT [MD = 3.85, 95%CrI (0.59, 7.10)], TQHXT vs. Control [MD = −3.03, 95%CrI (−5.26, −0.84)], XFZYT vs. Control [MD = −4.27, 95%CrI (−6.78, −1.78)], For Efficacy, MHFZXXT vs. Control [OR = 4.71, 95%CrI (2.64, 9.46)], SPT vs. Control [OR = 3.53, 95%CrI (2.21, 5.93)], TQHXT vs. Control [OR = 7.72, 95%CrI (4.60, 14.30)], WZYT vs. Control [OR = 3.70, 95%CrI (1.44, 10.19)], XFZYT vs. Control [OR = 4.80, 95%CrI (3.16, 7.28)], YXPGT vs. Control [OR = 4.42, 95%CrI (1.88, 11.24)]. For adverse events: MHFZXXT vs. Control [OR = 1.04, 95%CrI (0.12, 10.10)], TQHXT vs. Control [OR = 0.59, 95%CrI (0.09, 4.79)].

**TABLE 1:** Pairwise comparison of meta-analysis results.

Outcomes	Pairwise meta-analysis	No of study	Heterogeneity (%)	MD 95%CrI
Visual analogue scale	Control vs. BXBZTMT	3	99.8	0.79 (−0.86, 2.43)
	MHFZXXT vs. Control	5	95.4	−1.52 (−2.81, −0.24)
	SPT vs. Control	6	97.9	−2.87 (−4.21, −1.73)
	TQHXT vs. Control	6	96.3	−1.37 (−2.56, −0.20)
	WZYT vs. Control	1	NA	−1.45 (−4.90, 1.98)
	XFZYT vs. Control	5	98.5	−1.08 (−2.36, 0.20)
	YXPGT vs. Control	4	96.4	−1.00 (−2.41, 0.43)
Headache frequency	Control vs. BXBZTMT	3	99.7	1.11 (−0.06, 2.30)
	MHFZXXT vs. Control	3	99.8	−1.08 (−2.25, 0.09)
	SPT vs. Control	4	98.0	−1.54 (−2.56, −0.53)
	TQHXT vs. Control	8	98.2	−1.63 (−2.37, −0.91)
	WZYT vs. Control	5	19.0	−0.72 (−1.72, 0.31)
	XFZYT vs. Control	13	99.0	−1.70 (−2.28, −1.12)
	YXPGT vs. Control	5	96.9	−0.74 (−1.66, 0.18)
Duration of headache	Control vs. BXBZTMT	4	99.8	3.85 (0.59, 7.10)
	MHFZXXT vs. Control	3	95.8	−1.13 (−4.89, 2.59)
	SPT vs. Control	3	98.0	−2.89 (−6.64, 0.87)
	TQHXT vs. Control	9	99.3	−3.03 (−5.26, −0.84)
	WZYT vs. Control	3	0	−2.89 (−6.90, 1.15)
	XFZYT vs. Control	8	99.9	−4.27 (-6.78, −1.78)
	YXPGT vs. Control	5	98	−0.97 (-3.87, 1.93)
Efficacy	Control vs. BXBZTMT	6	31.9	0.18 (0.10, 0.33)
	MHFZXXT vs. Control	5	0	4.71 (2.64, 9.46)
	SPT vs. Control	8	0	3.53 (2.21, 5.93)
	TQHXT vs. Control	9	0	7.72 (4.60, 14.30)
	WZYT vs. Control	3	0	3.70 (1.44, 10.19)
	XFZYT vs. Control	12	0	4.80 (3.16, 7.28)
	YXPGT vs. Control	3	0	4.42 (1.88, 11.24)
Adverse events	Control vs. BXBZTMT	4	34.2	1.44 (0.20, 10.60)
	MHFZXXT vs. Control	3	79.4	1.04 (0.12, 10.10)
	SPT vs. Control	5	59.6	0.244 (0.04, 1.15)
	TQHXT vs. Control	4	65.6	0.59 (0.09, 4.79)
	WZYT vs. Control	2	0	0.91 (0.057, 14.70)
	XFZYT vs. Control	3	50.9	0.12 (0.01, 0.96)
	YXPGT vs. Control	3	0	0.18 (0.02, 1.51)

### Visual analogue scale

**FIGURE 2:**
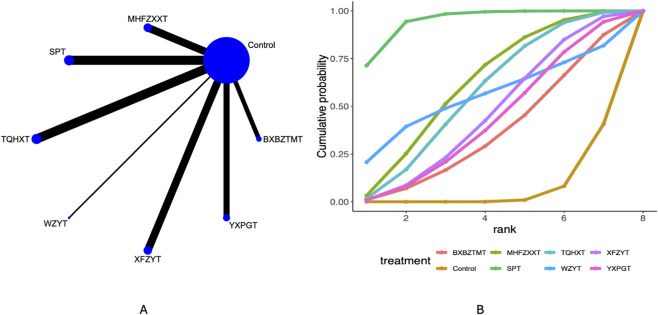
Visual analogue scale network meta-analysis results (**(A)** network diagram, **(B)** cumulative probability ranking diagram).

30 studies mentioned the visual analogue scale. Network plots (Figure 2A) indicate direct comparisons between YXPGT, BXBZTMT, WZYT, SPT, XFZYT, TQHXT, MHFZXXT, and Control. League tables (Supplementary Table S7) indicate that compared to Control, MHFZXXT [MD = −1.52, 95%CrI (−2.8, −0.25)], SPT [MD = −2.86, 95%CrI (−4.2, −1.72)], TQHXT [MD = −1.38, 95%CrI (−2.55, −0.19)] reduced the visual analogue scale in migraine patients. SPT demonstrated may be associated with greater improvement efficacy compared to XFZYT [MD = −1.77, 95%CrI (−3.65, −0.11)], YXPGT [MD = −1.87, 95%CrI (−3.88, −0.09)]. Cumulative probability curve ranking (Table 2; Figure 2B) indicated SPT (94.78%) > MHFZXXT (61.77%) > TQHXT (56.74%) > Control (7.13%).

**TABLE 2:** Ranking results under the cumulative probability curve.

Treatment	Visual analogue scale (%)	Headache frequency (%)	Duration of headache (%)	Efficacy (%)	Adverse events (%)
BXBZTMT	36.12	50.87	73.26	66.33	34.00
Control	7.13	2.90	9.51	0.01	13.22
MHFZXXT	61.77	49.62	31.21	60.11	29.22
SPT	94.78	71.87	57.86	34.42	68.95
TQHXT	56.74	77.53	61.20	91.02	58.10
WZYT	55.00	32.55	58.13	39.53	24.72
XFZYT	45.98	81.66	81.44	57.18	87.71
YXPGT	42.47	33.01	27.40	51.37	84.10

### Headache frequency

**FIGURE 3:**
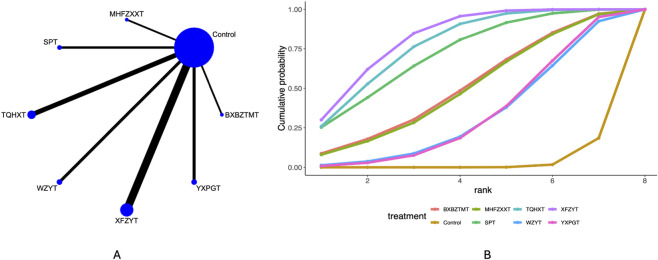
Headache frequency network meta-analysis results (**(A)** network diagram, **(B)** cumulative probability ranking diagram).

41 studies mentioned the headache frequency. Network plots (Figure 3A) indicate direct comparisons between YXPGT, BXBZTMT, WZYT, SPT, XFZYT, TQHXT, MHFZXXT, and Control. League tables (Supplementary Table S8) indicate that compared to Control, XFZYT [MD = −1.69, 95%CrI (−2.28, −1.13)], SPT [MD = −1.54, 95%CrI (−2.56, −0.52)], TQHXT [MD = −1.63, 95%CrI (−2.36, −0.91)] reduced the headache frequency in migraine patients. There were no significant differences between the various decoctions. Cumulative probability curve ranking (Table 2; Figure 3B) indicated XFZYT (81.66%) > TQHXT (77.53%) > SPT (71.87%) > Control (2.90%).

### Duration of headache

**FIGURE 4:**
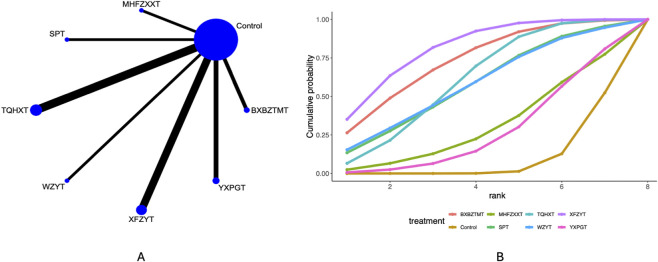
Duration of headache network meta-analysis results (**(A)** network diagram, **(B)** cumulative probability ranking diagram).

35 studies mentioned the duration of headache. Network plots (Figure 4A) indicate direct comparisons between YXPGT, BXBZTMT, WZYT, SPT, XFZYT, TQHXT, MHFZXXT, and Control. League tables (Supplementary Table S9) indicate that compared to Control, BXBZTMT [MD = −3.84, 95%CrI (−7.11, −0.62)], TQHXT [MD = −3.03, 95%CrI (−5.27, −0.83)], XFZYT [MD = −4.29, 95%CrI (−6.79, −1.79)] reduced the headache frequency in migraine patients. There were no significant differences between the various decoctions. Cumulative probability curve ranking (Table 2; Figure 4B) indicated XFZYT (81.44%) > TQHXT (61.20%) > WZYT (58.13%) > Control (9.15%).

### Efficacy

**FIGURE 5:**
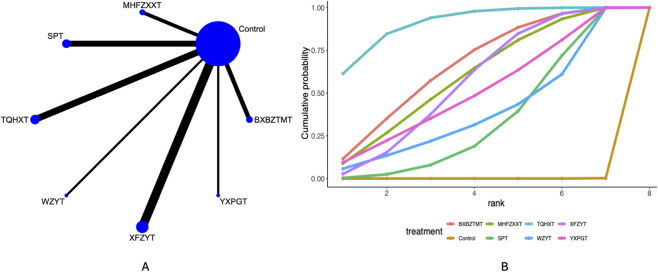
Efficacy network meta-analysis results (**(A)** network diagram, **(B)** cumulative probability ranking diagram).

46 studies mentioned the efficacy. Network plots (Figure 5A) indicate direct comparisons between YXPGT, BXBZTMT, WZYT, SPT, XFZYT, TQHXT, MHFZXXT, and Control. League tables (Supplementary Table S10) indicate that compared to Control, BXBZTMT [MD = 5.33 (3.05, 9.68)], MHFZXXT [OR = 4.71, 95%CrI (2.64, 9.46)], SPT [OR = 3.53, 95%CrI (2.21, 5.93)], TQHXT [OR = 7.72, 95%CrI (4.60, 14.30)], WZYT [OR = 3.70, 95%CrI (1.44, 10.19)], XFZYT [OR = 4.80, 95%CrI (3.16, 7.28)], YXPGT [OR = 4.42, 95%CrI (1.88, 11.24)] improved the efficacy in migraine patients. TSPT demonstrated inferior efficacy compared to TQHXT [OR = 0.46 (0.23, 0.94)]. Cumulative probability curve ranking (Table 2; Figure 5B) indicated TQHXT (91.02%) > BXBZTMT (66.33%) > MHFZXXT (60.11%) > Control (0.01%).

### Adverse events

**FIGURE 6:**
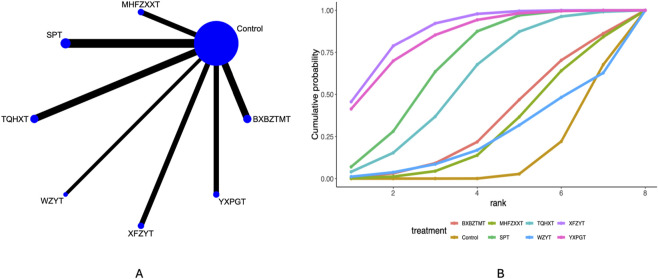
Adverse events network meta-analysis results (**(A)** network diagram, **(B)** cumulative probability ranking diagram).

24 studies mentioned the adverse events. Network plots (Figure 6A) indicate direct comparisons between YXPGT, BXBZTMT, WZYT, SPT, XFZYT, TQHXT, MHFZXXT, and Control. League tables (Supplementary Table S11) indicate that compared to XFZYT, MHFZXXT Will increase adverse events [OR = 3.9 (1.12, 14.45)]. Cumulative probability curve ranking (Table 2; Figure 6B) indicated XFZYT (87.71%) > YXPGT (84.10%) > SPT (68.95%) > Control (13.22%).

### Publication bias

This study detected publication bias using funnel plots. The analysis results (Supplementary Figures S2–S6) indicate that all metrics are symmetrical, suggesting a low likelihood of publication bias.

## Discussion

A recent network meta-analysis on nutraceuticals showed that certain nutraceuticals ranked highest for improving migraine characteristics (Ketata and Ellouz, 2025), To date, no network meta-analysis has specifically evaluated Chinese botanical drug decoctions for migraine. The present results suggest that most botanical drug decoctions may be associated with improvements in migraine symptoms compared with control conditions. However, differences between formulations varied across outcomes, and the overall certainty of evidence was moderate, requiring cautious interpretation.

In terms of pain intensity, SPT was associated with greater reductions in visual analogue scale scores compared with control and some other botanical drug decoctions (XFZYT and YXPGT), and ranked highest based on SUCRA probabilities (Lee et al., 2025). These findings suggest that SPT may be associated with improvements in acute pain outcomes. From a traditional medicine perspective, SPT is commonly used within a theoretical framework involving regulation of functional imbalances (Mao et al., 2022; Yang et al., 2024). Pharmacological studies indicate that metabolites derived from its constituent botanical drugs may exert analgesic effects through modulation of neurotransmitter release and inhibition of neurogenic inflammation (Li et al., 2023).

Regarding headache frequency, XFZYT, TQHXT, and SPT all significantly reduced migraine attacks, with no statistically significant differences observed among them. XFZYT ranked highest in cumulative probability sorting, suggesting a potential advantage in preventing recurrent migraine episodes (Chang et al., 2014). The method of promoting blood circulation and removing blood stasis is considered a key therapeutic principle for refractory or recurrent headaches. XFZYT may play a pivotal role in reducing migraine frequency by improving cerebral microcirculation, inhibiting platelet aggregation, and modulating inflammatory cytokine levels (Wu et al., 2016).

Regarding headache duration, XFZYT, TQHXT, and BXBZTMT all significantly shortened headache duration, with XFZYT exhibiting the highest-ranking probability. This finding further supports the potential advantage of blood-activating and stasis-resolving, meridian-unblocking and pain-relieving formulas in improving the overall course of migraine (Zhang et al., 2023). Notably, while certain decoctions demonstrated outstanding efficacy in shortening headache duration, differences between decoctions did not reach statistical significance (Zhou et al., 2024). This suggests clinical efficacy may be influenced by patient constitution, syndrome differentiation variations, and adjunctive therapies.

Regarding overall efficacy, all included Chinese botanical drug decoctions significantly outperformed the control treatment. TQHXT exhibited the most favorable comprehensive efficacy and ranked first in cumulative probability ordering. TQHXT, primarily indicated for treating ‘blood stasis obstructing the clear orifices’ syndrome, demonstrated overall advantages in alleviating headache severity, attack frequency, and associated symptoms (Li et al., 2024; Yuan et al., 2022). Results further indicated that SPT was inferior to TQHXT in overall efficacy, suggesting that while SPT excelled in relieving pain intensity, its comprehensive therapeutic effect may be inferior to formulas centered on the core therapeutic principle of ‘unblocking orifices and activating blood circulation’ (Gu et al., 2025; Mo et al., 2020).

Regarding safety, most Chinese botanical drug decoctions exhibited low adverse event rates and overall favorable safety profiles. XFZYT demonstrated the ranked highest (SUCRA-based) performance in adverse event ranking, whereas MHFZXXT potentially increased the risk of adverse events compared to XFZYT. This outcome may relate to MHFZXXT’s inclusion of pungent-warm yang-assisting herbs such as ephedra and aconite. While improving cold-pattern headaches, these ingredients may also heighten risks of adverse reactions like palpitations and insomnia (Ding et al., 2024; Rong et al., 2016). Consequently, clinical application must strictly adhere to indications while monitoring patients’ underlying conditions and tolerance.

### Strengths and limitations

This study employed a Bayesian network meta-analysis methodology to conduct a systematic and comprehensive comparison of the efficacy and safety of multiple Chinese botanical drug decoctions in migraine treatment. This approach integrates both direct and indirect evidence in the absence of direct head-to-head trials, thereby enabling a more accurate assessment of the relative effectiveness among different interventions. The study incorporated a comprehensive range of outcome measures, including pain intensity, headache frequency, headache duration, overall efficacy, and adverse events. This multidimensional approach facilitates a holistic assessment of the integrated therapeutic effects of Chinese botanical drug decoctions on migraine. Furthermore, the cumulative probability ranking methodology provided a ranked comparison of the various formulations, offering clinicians more valuable evidence-based guidance for selecting appropriate botanical drug decoctions based on syndrome differentiation and treatment principles.

This study nevertheless exhibits certain limitations. Firstly, the overall quality of included studies varied considerably, with inadequate reporting of randomization methods, allocation concealment, and blinding protocols in some literature, potentially increasing the risk of bias. Secondly, discrepancies existed across studies regarding migraine diagnostic criteria, syndrome distribution, disease duration, and concomitant medication use, which may have impacted result stability. Moreover, the predominantly short follow-up periods in most studies hindered assessment of the long-term preventive efficacy and long-term safety of Chinese botanical drug decoctions for migraine. Finally, the absence of in-depth analyses based on syndrome patterns or patient subgroups limited the applicability of findings to individualized treatment. A further important limitation relates to the reporting quality and reproducibility of the herbal interventions. As shown in Supplementary Table S3, many included studies did not adequately report the formula provenance, full ingredient composition, taxonomically valid relevant potential species, or preparation procedures. As a result, interventions carrying the same decoction name may not necessarily represent the same pharmacologically comparable preparation across studies. This limits interpretability, weakens confidence in cross-study comparison, and reduces the reproducibility of the evidence base. Accordingly, the findings of this review should be interpreted with caution, not only because of the methodological limitations of the clinical trials, but also because the herbal interventions themselves were often insufficiently characterized. Future trials should report formula origin, complete composition, taxonomically validated relevant potential species, plant part/medicinal material identity, and preparation details in a standardized manner.

### Clinical significance and future research directions

This study suggests that botanical drug decoctions may be considered as potential complementary approaches for migraine management, particularly in patients who do not respond adequately or experience adverse effects with standard biomedical treatments. However, given the variability in study quality and indirect comparisons, these findings should be interpreted cautiously.

Future research should focus on well-designed, multicenter randomized controlled trials with standardized diagnostic criteria and outcome measures. In addition, further studies integrating pharmacological investigations of active metabolites and clinical evidence are needed to better understand the mechanisms and optimize the clinical application of botanical drug decoctions.

## Conclusion

The findings of this study indicate that most Chinese botanical drug decoctions demonstrated showed potential benefits over control treatments in reducing pain intensity, decreasing headache frequency, and improving overall therapeutic efficacy.

Among the evaluated interventions, SPT showed the ranked highest (SUCRA-based) performance in pain relief, XFZYT demonstrated advantages in reducing attack frequency and duration, and TQHXT ranked highest in overall efficacy.

## Data Availability

The original contributions presented in the study are included in the article/Supplementary Material, further inquiries can be directed to the corresponding author.
