# Supplementary material for: Efficacy and safety of Chinese botanical drug decoctions for migraine: a Bayesian network meta-analysis
Source: Front Pharmacol. 2026 May 1;17:1799920. doi: 10.3389/fphar.2026.1799920 (PMC13175960; doi:10.3389/fphar.2026.1799920)
Supplement: Supplementary file 2 [file DataSheet2.docx]

Table S1 search strategy

(("Migraine Disorders"[Mesh]) OR (((((((((((((((((((((((((((((((((((Migraine Disorders[Title/Abstract]) OR (Disorder, Migraine[Title/Abstract])) OR (Disorders, Migraine[Title/Abstract])) OR (Migraine Disorder[Title/Abstract])) OR (Headache, Migraine[Title/Abstract])) OR (Headaches, Migraine[Title/Abstract])) OR (Migraine Headaches[Title/Abstract])) OR (Migraine[Title/Abstract])) OR (Migraines[Title/Abstract])) OR (Migraine Headache[Title/Abstract])) OR (Acute Confusional Migraine[Title/Abstract])) OR (Acute Confusional Migraines[Title/Abstract])) OR (Migraine, Acute Confusional[Title/Abstract])) OR (Migraines, Acute Confusional[Title/Abstract])) OR (Status Migrainosus[Title/Abstract])) OR (Abdominal Migraine[Title/Abstract])) OR (Abdominal Migraines[Title/Abstract])) OR (Migraine, Abdominal[Title/Abstract])) OR (Migraines, Abdominal[Title/Abstract])) OR (Cervical Migraine Syndrome[Title/Abstract])) OR (Cervical Migraine Syndromes[Title/Abstract])) OR (Migraine Syndrome, Cervical[Title/Abstract])) OR (Migraine Syndromes, Cervical[Title/Abstract])) OR (Hemicrania Migraine[Title/Abstract])) OR (Hemicrania Migraines[Title/Abstract])) OR (Migraine, Hemicrania[Title/Abstract])) OR (Migraines, Hemicrania[Title/Abstract])) OR (Migraine Variant[Title/Abstract])) OR (Migraine Variants[Title/Abstract])) OR (Variant, Migraine[Title/Abstract])) OR (Variants, Migraine[Title/Abstract])) OR (Sick Headache[Title/Abstract])) OR (Headache, Sick[Title/Abstract])) OR (Headaches, Sick[Title/Abstract])) OR (Sick Headaches[Title/Abstract]))) AND (("juzentaihoto" [Supplementary Concept]) OR ((Tang[Title/Abstract]) OR (Decoction[Title/Abstract])))

Table S2 Basic characteristics of included studies

| Author | Year | Sample size | Gender(M/F) | Mean age | Intervention | Follow-up | Outcomes |
| --- | --- | --- | --- | --- | --- | --- | --- |
| R Liu | 2020 | YXPGT:38  Control:38 | 29/47 | YXPGT:38.67  Control:38.64 | YXPGT:Twice/day;1 month | NR | F2;F3;F5 |
| XY Wu | 2019 | YXPGT:50  Control:50 | 48/52 | YXPGT:37.95  Control:37.79 | YXPGT:Twice/day;2month | NR | F1;F2;F3 |
| SX Zhang | 2023 | YXPGT:62  Control:62 | 48/72 | YXPGT:39.47  Control:38.92 | YXPGT:Twice/day;4 weeks | NR | F1;F2;F3;F4 |
| QX Zhang | 2019 | YXPGT:32  Control:32 | 17/47 | YXPGT:49.86  Control:50.23 | YXPGT:Twice/day;4 weeks | NR | F1;F2;F3;F4;F5 |
| YF Wang | 2019 | YXPGT:39  Control:39 | 22/56 | YXPGT:38.54  Control:38.76 | YXPGT:Twice/day;2 month | NR | F1;F2;F3;F4 |
| DY Li | 2020 | BXBZTMT: 42  Control:42 | 35/49 | BXBZTMT:47.56  Control:47.25 | BXBZTMT:Twice/day;2 weeks | NR | F2;F3;F4;F5 |
| F Yang | 2022 | BXBZTMT: 60  Control:60 | 28/92 | BXBZTMT:52.38  Control:52.69 | BXBZTMT:Twice/day;2 weeks | 3 month | F1;F3;F4 |
| JH Ou | 2016 | BXBZTMT: 45  Control:45 | 38/52 | BXBZTMT:40  Control:41 | BXBZTMT:Three times a day;6 weeks | NR | F4;F5 |
| QC Zheng | 2021 | BXBZTMT: 75  Control:75 | 38/112 | BXBZTMT:34.58  Control:34.72 | BXBZTMT:Twice/day;4 weeks | NR | F1;F2;F3;F4;F5 |
| XY Guo | 2013 | BXBZTMT: 32  Control:31 | 27/36 | BXBZTMT:34.8  Control:33.9 | BXBZTMT:Three times a day;15 days | NR | F1;F2;F3;F4 |
| SM Chen | 2003 | BXBZTMT: 72  Control:35 | 28/79 | BXBZTMT:39.8  Control:37.6 | BXBZTMT:Twice/day;2 weeks | 1 year | F4;F5 |
| HW Liu | 2022 | WZYT: 40  Control:41 | 26/55 | WZYT: 40.25  Control:40.46 | WZYT:Twice/day;12 weeks | 4 weeks | F2;F3 |
| HY Liu | 2006 | WZYT: 32  Control:30 | 28/34 | WZYT: 34  Control:30 | WZYT:Three times a day;4 weeks | 1 year | F2;F3;F4 |
| HL Zhang | 2024 | WZYT: 30  Control:30 | 23/37 | WZYT: 40.41  Control:40.26 | WZYT:Three times a day,12 weeks | NR | F2;F3;F4;F5 |
| ZJ Li | 2019 | WZYT: 40  Control:40 | 31/49 | WZYT:31.8  Control:30.7 | WZYT:Three times a day,8 weeks | 4 weeks | F1;F2;F4 |
| HW Liu | 2018 | WZYT: 51  Control:27 | 14/51 | WZYT:39.78  Control:35.72 | WZYT:Twice/day;12 weeks | 4 weeks | F2;F5 |
| XH Ge | 2023 | SPT: 40  Control:40 | 36/44 | SPT:40.76  Control:40.83 | SPT:Twice/day;3 month | NR | F1;F2;F3;F4;F5 |
| LL Li | 2025 | SPT: 57  Control:56 | 54/59 | SPT:48.02  Control:48.59 | SPT:Twice/day;4 weeks | NR | F1;F4 |
| RJ Li | 2013 | SPT: 50  Control:50 | 37/63 | SPT:33.9  Control:34.6 | SPT:Twice/day;3 month | 6 month | F1;F2;F4;F5 |
| H Li | 2012 | SPT: 50  Control:50 | 37/63 | SPT:33.9  Control:34.6 | SPT:Twice/day;14days | NR | F4 |
| ZK Liang | 2011 | SPT: 28  Control:32 | 21/39 | SPT:33.2  Control:32.7 | SPT:Twice/day;30days | 1 year | F4;F5 |
| B Shen | 2016 | SPT: 58  Control:57 | 40/75 | SPT:26.98  Control:25.54 | SPT:Twice/day;4 weeks | 4 weeks | F1;F2;F3;F4;F5 |
| HB Gu | 2023 | SPT: 51  Control:52 | 29/74 | SPT: 36.62  Control:43.43 | SPT:Once/day;4 weeks | 3 month | F1;F2;F3;F4;F5 |
| KX Ma | 2025 | SPT: 30  Control:30 | 17/43 | SPT: 31.13  Control:32.87 | SPT:Twice/day;4 weeks | NR | F1;F4 |
| HQ Wan | 2014 | XFZYT: 56  Control:56 | 59/73 | XFZYT: 35.8  Control:34.25 | XFZYT:Twice/day;30days | NR | F1;F2;F4 |
| YH Liu | 2015 | XFZYT: 38  Control:31 | 40/29 | XFZYT: 38.76  Control:39.34 | XFZYT:Twice/day;30days | NR | F2;F3;F4 |
| F Liu | 2018 | XFZYT: 61  Control:61 | 49/73 | XFZYT: 37.05  Control:36.29 | XFZYT:Once/day;30days | NR | F2;F4 |
| HY Shi | 2023 | XFZYT: 46  Control:46 | 27/65 | XFZYT: 46.29  Control:45.84 | XFZYT:Twice/day;45days | 2 month | F1;F2;F3;F4 |
| B Zhang | 2011 | XFZYT: 25  Control:25 | 23/27 | XFZYT: 30.5  Control:33.6 | XFZYT:Twice/day;one month | NR | F4 |
| L Cao | 2015 | XFZYT: 45  Control:45 | 24/66 | XFZYT: 26.9  Control:27.2 | XFZYT:Three times a day;2 weeks | 1 year | F4;F5 |
| B li | 2018 | XFZYT: 30  Control:30 | 18/42 | XFZYT: 31.33  Control:31.05 | XFZYT:Twice/day | NR | F2;F3;F5 |
| ZS Li | 2025 | XFZYT: 45  Control:45 | 26/64 | XFZYT: 52.6  Control:51.3 | XFZYT:Twice/day;4 weeks | NR | F2;F4 |
| P Wang | 2016 | XFZYT: 30  Control:30 | 18/42 | XFZYT: 30.76  Control:30.89 | XFZYT:Twice/day;21 days | NR | F1;F2;F3 |
| YX Bai | 2023 | XFZYT: 50  Control:50 | 41/59 | XFZYT: 30.76  Control:30.89 | XFZYT:Twice/day;4 weeks | NR | F1;F2;F3;F4 |
| LR Cui | 2017 | XFZYT: 50  Control:50 | 57/43 | XFZYT: 45.6  Control:45.3 | XFZYT:Once/day;20 days | NR | F1;F2 |
| YS Ma | 2016 | XFZYT: 40  Control:40 | 30/50 | XFZYT: 32.6  Control:32.5 | XFZYT:Twice/day;2 weeks | NR | F2;F3;F4;F5 |
| P Wei | 2015 | XFZYT: 40  Control:40 | 23/57 | XFZYT: 31.8  Control:33.5 | XFZYT:Once/day;20 days | 1 year | F2;F4 |
| WX Li | 2016 | XFZYT: 40  Control:40 | 30/50 | XFZYT: 32.6  Control:32.5 | XFZYT:Twice/day | NR | F2;F3;F4 |
| TH Li | 2016 | XFZYT: 39  Control:39 | 29/49 | XFZYT: 47.3  Control:46.3 | XFZYT:Twice/day;8 days | NR | F2;F3;F4 |
| S Ye | 2023 | TQHXT: 40  Control:40 | 22/58 | TQHXT: 42.37  Control:42.59 | TQHXT:Twice/day; 6 weeks | 6 month | F1;F4 |
| QL Zhang | 2021 | TQHXT: 43  Control:43 | 17/69 | TQHXT: 34.77  Control:35.34 | TQHXT:Twice/day; 3 weeks | NR | F1;F3;F4 |
| J Li | 2016 | TQHXT: 34  Control:34 | 39/29 | TQHXT: 46.40  Control:46.58 | TQHXT:Twice/day; 2 weeks | NR | F1;F2;F3;F4 |
| SC Liang | 2021 | TQHXT: 60  Control:60 | 84/36 | TQHXT: 65  Control:65 | TQHXT:Twice/day; 1 weeks | NR | F1;F2;F3;F5 |
| Y Wang | 2017 | TQHXT: 24  Control:24 | 13/35 | TQHXT: 45.33  Control:45.22 | TQHXT:Twice/day; 28 days | 3 month | F4 |
| CC Su | 2015 | TQHXT: 34  Control:34 | 34/34 |  | TQHXT:Once/day;3 month | 6 month | F2;F3;F4 |
| YX Zhao | 2023 | TQHXT: 40  Control:40 | 29/51 | TQHXT: 33.10  Control:32.83 | TQHXT:Once/day;4 weeks | NR | F2;F3;F5 |
| JQ Guo | 2023 | TQHXT: 36  Control:36 | 33/39 | TQHXT: 46.83  Control:47.26 | TQHXT:Twice/day; 4 weeks | 3 month | F1;F2;F3;F4;F5 |
| YL Qian | 2006 | TQHXT: 57  Control:60 | 47/70 | TQHXT: 42.5  Control:41.5 | TQHXT:Twice/day; 28 days | NR | F2;F3;F4 |
| SS Chen | 2014 | TQHXT: 47  Control:40 | 53/34 | TQHXT: 44.8  Control:43.2 | TQHXT:Twice/day; 4 weeks | 1 year | F2;F3;F4 |
| JN Gao | 2019 | TQHXT: 20  Control:20 | 23/17 | TQHXT: 36.28  Control:35.92 | TQHXT:Twice/day; 2 weeks | NR | F1;F2;F3;F4;F5 |
| H Fu | 2020 | MHFZXXT: 46  Control:46 | 45/47 | MHFZXXT: 39.1  Control:38.5 | MHFZXXT: Twice/day; 1 month | NR | F1;F4 |
| Y Liu | 2022 | MHFZXXT: 40  Control:40 | 49/31 | MHFZXXT: 45.44  Control:45.85 | MHFZXXT: Twice/day; 6 weeks | NR | F1;F2;F3;F4 |
| D Li | 2019 | MHFZXXT: 47  Control:46 | 53/40 | MHFZXXT: 35.90  Control:36.10 | MHFZXXT: Twice/day; 2weeks | NR | F1 |
| GJ Yang | 2019 | MHFZXXT: 45  Control:45 | 38/52 | MHFZXXT: 57.20  Control:56.6 | MHFZXXT: Once/day; 3 month | NR | F2;F3;F4;F5 |
| JD Lin | 2024 | MHFZXXT: 43  Control:43 | 22/64 | MHFZXXT: 44.14  Control:43.55 | MHFZXXT: Twice/day; 1 month | NR | F1;F4;F5 |
| YM Luo | 2021 | MHFZXXT: 32  Control:33 | 23/42 | MHFZXXT: 46.46  Control:46.28 | MHFZXXT: Once/day; 1 month | NR | F2;F3 |
| BH Zhao | 2023 | MHFZXXT: 46  Control:45 | 22/69 | MHFZXXT: 42.19  Control:42.25 | MHFZXXT: Twice/day; 1 month | NR | F1;F4;F5 |

FYXPGT: Yangxue Pinggan Decoction; BXBZTMT: Banxia Baizhu Tianma Decoction; WZYT: Wuzhuyu Decoction; SPT: Sanpian Decoction; XFZYT: Xuefu Zhuyu Decoction; TQHXT: Tongqiao Huoxue decoction; MHFZXXT: Mahuang Fuzi Asarum Decoction; NR: not reported; F1: visual analogue scale; F2: headache frequency; F3: Duration of headache; F4: Efficacy; F5: Adverse events

(1-57)

1. Li Tuanhui. Observation on the Therapeutic Effect of Modified Xuefu Zhuyu Decoction Combined with Flunarizine for Migraine. Medical Information. 2016;29(34):294-5.

2. Li Weixiong, Zou Weidong, Liu Qinbiao, Xie Dongfeng. Clinical Study on the Combined Use of Xuefu Zhuyu Decoction and Western Medication for Migraine. Northern Pharmacy. 2016;13(4):50-51.

3. Wei Ping, Xi Heng. Treatment of 40 Cases of Migraine with Modified Xuefu Zhuyu Decoction. Western Chinese Medicine. 2015;28(12):89-90.

4. Gao Junning, Yang Fei, Wei Yan. Observation on the Efficacy of Tongqiao Huoxue Decoction Combined with Wrist-Ankle Acupuncture in Treating Migraine. Hainan Medicine. 2019;30(16):2113-6.

5. Ma Yongsheng. Observation on the Efficacy of Xuefu Zhuyu Decoction Combined with Western Medication in Treating 40 Cases of Primary Migraine. Journal of Hebei North University (Natural Science Edition). 2016;32(8):26-7,9.

6. Ma Kaixuan, Fan Ruidong, Yuan Ping, Feng Kai. Clinical observation of Huacai Sanpian Tang in treating migraine. Capital Food and Medicine. 2025;32(14):160-3.

7. Gu Hongbing, Zhang Pingping, Zhao Yuqi, Xu Jialong, Chen Jindan. Clinical Study on Acupuncture Combined with Sanpian Decoction for Migraine. New Chinese Medicine. 2023;55(1):169-73.

8. Huo Lirong, Zhou Ping. Efficacy of Modified Blood Residence Stasis-Expelling Decoction in Treating Acute Migraine and Its Effects on Serum ICAM-1 and IL-6 Levels. Sichuan Journal of Traditional Chinese Medicine. 2017;35(4):124-6.

9. Chen Shanshan. Modified Unblocking Orifices and Activating Blood Decoction in Treating 47 Cases of Migraine. Medical Frontier. 2014(34):338-9.

10. Chen Shaomei. Therapeutic efficacy of Sanchong Banxia Baizhu Tianma Decoction in 72 cases of migraine. Chinese Journal of Traditional Chinese Medicine Emergency. 2003;12(2).

11. Qian Yuliang, Yan Dong. Clinical observation of Tongqiao Huoxue Decoction in 57 cases of migraine. Hunan Journal of Traditional Chinese Medicine. 2006;22(6):6-8.

12. Guo Jinqiao, Lu Chunqian. Clinical efficacy of modified Tongqiao Huoxue Decoction combined with flunarizine in treating vestibular migraine. Shenzhen Journal of Integrated Traditional Chinese and Western Medicine. 2023;33(9):52-5.

13. Guo Xiaoyun. Clinical Observation of 32 Cases of Migraine Treated with Modified Pinellia, Atractylodes and Gastrodia Decoction. Yunnan Journal of Traditional Chinese Medicine and Chinese Medicine. 2013;34(11).

14. Zheng Quancheng, Liu Jianhao, Zhang Yu, Zeng Linghui, Huang Xinyi, Fu Yongjun. Observation on the Combined Use of Modified Pinellia, Atractylodes and Gastrodia Decoction and Acupuncture for Migraine with Wind-Phlegm Disturbing the Upper Burner. Chinese Journal of Experimental Formulary. 2021;27(3):111-6.

15. Zhao Bohua, Wang Zhenyao, Yang Xiaowei. Clinical Study on Ephedra, Aconite and Asarum Decoction Combined with Balanced Acupuncture for Migraine with Yang Deficiency and Cold Aggregation Pattern. International Journal of Chinese Medicine and Pharmacy. 2023;45(6):699-702.

16. Zhao Yunxia. Study on the Treatment of Migraine with Warm Acupuncture Combined with Modified Tongqiao Huoxue Decoction. Heilongjiang Medical Science. 2023;46(3).

17. Su Chengcai. Observation on the Efficacy of Modified Tongqiao Huoxue Decoction in Treating 68 Cases of Migraine. Chinese Journal of Continuing Medical Education. 2015;7(25).

18. Luo Yumei. Efficacy comparison of Ephedra-Aconite-Asarum Decoction combined with Headache Relief Capsules for migraine patients. Nutrition and Health. 2021(30):113-4.

19. Bai Yuexiu, Fu Xishun. Clinical observation on the treatment of blood stasis obstructing collaterals type migraine with Modified Blood Residence Stasis-Resolving Decoction combined with Ascending-Descending Powder. Chinese Community Physician. 2023;39(2):81-3,6.

20. Shen Bin, Yu Chuan, Wang Lei, Liu Fuqi, Xu Yinping, Zou Yihui, et al. Treatment of 60 Cases of Liver Qi Stagnation with Blood Stasis Type Migraine Using Modified Sanpian Decoction Granules. Modern Distance Education of Traditional Chinese Medicine in China. 2016;14(4):51-4.

21. Wang Ying, Lin Hai. Randomised Parallel Controlled Study of Tongqiao Huoxue Decoction Combined with Flunarizine for Blood Stasis-Type Migraine. Journal of Practical Internal Medicine. 2017;31(3):54-6.

22. Wang Yanfeng. Observation on the Effect of Yangxue Pinggan Decoction Combined with Flunarizine in Treating Migraine. Journal of Practical Traditional Chinese Medicine. 2019;35(1):95-6.

23. Wang Ping. Clinical Observation on Modified Xuefu Zhuyu Decoction Combined with Nimodipine in Treating Migraine. Journal of Liaoning University of Traditional Chinese Medicine. 2016;18(3):180-3.

24. Ou Jianghong, Ablaiti Aizezi, Wan Xuemei. Efficacy of Modified Pinellia, Atractylodes and Gastrodia Decoction on Patients with Phlegm-Turbidity Disturbing the Upper Burner Type Migraine and Its Effects on Endothelin-1 and Nitric Oxide Levels. Journal of Hunan University of Chinese Medicine. 2016;36(7):65-7.

25. Liang Shucheng. Efficacy of Tongqiao Huoxue Decoction Combined with Wrist-Ankle Acupuncture in Treating Migraine. World Latest Medical Information Abstract (Continuous Electronic Journal). 2021;21(5):233-4.

26. Liang Zengkun, Yao Guozhou, Liang Xincai. Analysis of the Efficacy of Sanpian Decoction Combined with Electroacupuncture in Treating Migraine. Journal of Practical Traditional Chinese Medicine. 2011;27(6):366-8.

27. Lin Jiandong. Observation on the Efficacy of Balanced Acupuncture Combined with Ephedra-Aconite-Asarum Decoction and Flunarizine Hydrochloride in Treating Migraine. Heilongjiang Medicine. 2024;37(3).

28. Yang Ganjun, Zhang Xiaoqiang, Sun Yi. Efficacy of Dabu Yuanjian Decoction Combined with Ephedra-Aconite-Asarum Decoction in Treating Kidney-Deficiency Headache Pattern During Acute Migraine Episodes. Massage and Rehabilitation Medicine. 2019;10(10):37-9.

29. Yang Fan, Zhang Ling, Li Lejun, Li Shengrang, Yu Yalan. Observation on the Analgesic Efficacy of Modified Pinellia, Atractylodes and Gastrodia Decoction for Acute Migraine Attacks (Phlegm-Turbidity Pattern). Chinese Journal of Traditional Chinese Medicine Emergency. 2022;31(4):660-2.

30. Li Jing. Observation on the Effect of Tongqiao Huoxue Decoction Combined with Cibaline in Treating Blood-Stasis Pattern Migraine. International Journal of Medical and Health News. 2016;22 (5).

31. Li Hui, Li Ruijie, Li Fang. Randomised Controlled Clinical Study of Modified Sanpian Decoction for Migraine. Journal of Practical Internal Medicine. 2012;26(18):12-3.

32. Li Ruijie, Xi Xinjuan, Li Fang, Li Hui. Treatment of 50 Cases of Migraine with Modified Sanpian Decoction. Modern Chinese Medicine. 2013;33(1):13-5.

33. Li Zhijin, Wu Pingcai, Li Guozheng. Wu Zhu Yu Tang combined with warm acupuncture for treating cold-凝 blood stasis type migraine without aura. Journal of Traditional Chinese Medicine. 2019;34(12):2641-5.

34. Li Zhishen, Shi Hongyan, Yang Shaokun, Xun Junfeng, Zhou Guifu. Effects of Xuefu Zhuyu Decoction Combined with Cyproheptadine Hydrochloride Tablets on Cerebral Hemodynamics and Vasoactive Substances in Patients with Blood Stasis Obstructing Collaterals Migraine. Journal of Modern Chinese-Western Medicine Integration. 2025;34(11):1500-3,7.

35. Li Bin. Clinical value of modified Blood Residence Stasis-Expelling Decoction combined with Western medicine in treating primary headaches. Health Must-Read. 2018(24):31.

36. Li Dongyan. Observations on treating migraine with the Chinese herbal formula Pinellia, Atractylodes and Gastrodia Decoction. China Medical Guide. 2020;18(18).

37. Li Dan. Effects of Ephedra-Aconite-Asarum Decoction Combined with Linggui Bafa Acupuncture on VAS Scores and Quality of Life in Migraine Patients. Northern Pharmacy. 2019;16(1).

38. Li Lili, He Lili, Liu Yan. Clinical Efficacy Observation and Mechanism Exploration of Modified Sanpian Decoction Granules for Oral Administration in Migraine Treatment. Shandong Medicine. 2025;65(8):67-71.

39. Cao Li, Dai Yanping, Qi Dan, Jin Mei, Zhang Chunyuan, Li Jun. Clinical efficacy analysis of Tongxinluo Xuefu Zhuyu Decoction in treating migraine. Chinese and Foreign Medicine. 2015;34(04):112-3.

40. Ge Xuehua, Zhang Yuanxing, Shen Xiaoying. Clinical efficacy analysis of Chuanxiong San Pian Tang in treating phlegm-turbidity disturbing the upper burner type migraine. Kangyi. 2023(2):238-40.

41. Zhang Qiuxiang, Sun Fengxia. Clinical Efficacy of Yangxue Pinggan Decoction in Treating Hemicrania Comparsa and Its Effects on Serum ET-1, CGRP and Oral NO Levels. Sichuan Journal of Traditional Chinese Medicine. 2019;37(1):134-8.

42. Zhang Shuxian, Xiao Caixia. Clinical observation of Yangshen Pinggan Decoction combined with flunarizine in treating migraine. Journal of Practical Traditional Chinese Medicine. 2023;39(3):537-9.

43. Zhang Bo. Observation on the efficacy of modified Xuefu Zhuyu Decoction in treating migraine. Journal of Changchun University of Traditional Chinese Medicine. 2011; 27(3):424-5.

44. Zhang Qingli, Ren Wenqi. Treatment of 43 Cases of Aura-Free Migraine with Blood Stasis Pattern Using Blood-Letting Acupuncture Combined with Tongqiao Huoxue Decoction. Guangming Traditional Chinese Medicine. 2021;36(9):1471-4.

45. Zhang Honglin. Clinical efficacy analysis of modified Evodia Decoction combined with acupuncture at Shaoyang meridian points for cold-凝 blood stasis pattern in migraine. Weekly Digest · Elderly Care Weekly. 2024(12).

46. Shi Hongyan, Yang Shaokun, Xun Junfeng, Zhang Chenliu, Li Zhishen. Clinical observation of modified Blood Residence Stasis-Expelling Decoction combined with cyproheptadine hydrochloride tablets for treating blood stasis-type migraine. China Hospital Drug Evaluation and Analysis. 2023;23(12):1450-3.

47. Wu Xiongying. Observation on the Effect of Yangxue Pinggan Decoction in the Treatment of Refractory Migraine of Blood Deficiency with Liver Hyperactivity Pattern. Chinese and Foreign Medical Research. 2019;17(8).

48. Ye Sheng, Xu Tao, Zhang Qingchuan. Clinical Efficacy of Modified Tongqiao Huoxue Decoction Combined with Western Medication in Treating Blood Stasis-Type Migraine. Journal of Rational Clinical Drug Use. 2023;16(3):62-4.

49. Liu Fei. Clinical Study on the Treatment of Migraine with Modified Blood Residence Stasis-Expelling Decoction. E-Journal of Cardiovascular Diseases in Chinese and Western Medicine. 2018;6(21):152.

50. Liu Hongyan, Liu Chunyan. Clinical Observation of Wu Zhu Yu Tang in Treating 32 Cases of Migraine. Chinese Journal of Traditional Chinese Medicine Emergency. 2006;15(6):608,25.

51. Liu Rui. Clinical Efficacy Observation of Yang Xue Ping Gan Tang in Treating Migraine. Capital Food and Medicine. 2020;27(8).

52. Liu Yuhong. Clinical experience with Xuefu Zhuyu Decoction for treating migraine. Chinese Health Standards Management. 2015(11):105-6.

53. Liu Yan, Xu Ye. Clinical study on Ma Huang Fu Zi Xi Xin Decoction for treating migraine. Henan Journal of Traditional Chinese Medicine. 2022;42(10):1475-8.

54. Liu Hongwei, Wang Yizhan, Yuan Chen, Li Xin, Fu Caihong, Zhang Xiaojian. Clinical efficacy of modified Evodia Decoction combined with acupuncture at Shaoyang meridian points for migraine with cold-凝 blood stasis pattern. Liaoning Journal of Traditional Chinese Medicine. 2022;49(07):135-9.

55. Fu Hua, Xiang Fei. Clinical efficacy of Ephedra, Aconite and Asarum Decoction combined with flunarizine in treating migraine. Electronic Journal of Clinical Medicine Literature. 2020;7(59):151,3.

56. Wan HQ, Hu FY. Efficacy analysis of Modified Blood Residence Stasis-Expelling Decoction for migraine treatment. Journal of Yichun University. 2014;36(9):70-1.

57. Liu HW, Zou YH, Cao KG, Yu LH, Zhang Y, Fu CH, et al. Efficacy of Modified Wuzhuyu Decoction Granule ( ) for Migraine Patients with Cold and Stasis Obstructing Meridian Syndrome: a Randomised, Double-Blind, Placebo-Controlled Trial. Chinese Journal of Integrative Medicine. 2018;24(6):409-14.

Table S3 each decoction composition

| Study | Decoction name | Official pharmacopoeial/regulatory standard | Authoritative source / origin stated in article | Composition reported in the included study | Relevant potential species | Preparation / administration reported | Reporting quality assessment |
| --- | --- | --- | --- | --- | --- | --- | --- |
| Liu Rui_2020 | Yangxue Pinggan Decoction | Not identified in the study | Described as an empirical formula transmitted by Professor Guan Youbo | Gypsum Fibrosum 30 g; Rehmanniae Radix 15 g; Paeoniae Radix Alba 15 g; Haematitum 15 g; Inulae Flos 10 g; Angelicae Sinensis Radix 10 g; Chuanxiong Rhizoma 10 g; Polygoni Multiflori Caulis 10 g; Cyperi Rhizoma 10 g; Glycyrrhizae Radix et Rhizoma 5 g | Rehmannia glutinosa (Gaertn.) DC.; Paeonia lactiflora Pall.; Angelica sinensis (Oliv.) Diels; Ligusticum chuanxiong Hort.; Polygonum multiflorum Thunb. [stem drug, Polygoni Multiflori Caulis]; Cyperus rotundus L.; Glycyrrhiza uralensis Fisch. ex-DC. / G. inflata Bat. / G. glabra L.; mineral drugs also reported (Gypsum Fibrosum, Haematitum). | Soaked in water for 1 h; first decoction simmered for 30 min to obtain 200 mL; second decoction reduced from 400 mL to 200 mL; mixed and taken twice daily, morning and evening; 1 month of treatment | 2 = Limited |
| Wu Xiongying_2019 | Yangxue Pinggan Decoction | Not identified in the study | Not stated | Haliotidis Concha (Shijueming) 30 g; Margaritae Concha (Zhenzhumu) 30 g; Uncariae Ramulus cum Uncis 20 g; Gastrodiae Rhizoma 12 g; Chuanxiong Rhizoma 12 g; Angelicae Sinensis Radix 10 g; Tribuli Fructus 10 g; Rehmanniae Radix 10 g; Paeoniae Radix Alba 10 g; Angelicae Dahuricae Radix 12 g; optional additions: Gentianae Radix/Huangqin for liver fire, Gypsum/Zhimu for stomach fire, Chaihu/Lümeihua for qi stagnation, Chenpi/Banxia for phlegm, Taoren/Honghua for blood stasis | Haliotis spp. [for Haliotidis Concha], Pteria martensii (Dunker) [for Margaritifera/ Margaritae Concha as reported medicinal material], Uncaria rhynchophylla (Miq.) Miq. ex Havil. / related accepted Uncaria spp., Gastrodia elata Blume, Ligusticum chuanxiong Hort., Angelica sinensis (Oliv.) Diels, Tribulus terrestris L., Rehmannia glutinosa (Gaertn.) DC., Paeonia lactiflora Pall., and Angelica dahurica (Hoffm.) | Decoction concentrated to 300 mL; taken twice daily; 1 dose/day; treatment for 2 months | 2 = Limited |
| Zhang Shuxian_2023 | Yangxue Pinggan Decoction | Not identified in the study | Not stated | Haliotidis Concha 30 g; Margaritae Concha 30 g; Uncariae Ramulus cum Uncis 20 g; Haematitum 15 g; Gastrodiae Rhizoma 12 g; Chuanxiong Rhizoma 12 g; Angelicae Dahuricae Radix 12 g; Tribuli Fructus 10 g; Angelicae Sinensis Radix 10 g; Polygoni Multiflori Caulis 10 g; Rehmanniae Radix 15 g; Paeoniae Radix Alba 15 g; Glycyrrhizae Radix et Rhizoma 5 g; optional additions for liver fire, stomach fire, liver qi stagnation, phlegm dampness, and blood stasis | Haliotis spp., Pteria martensii (Dunker), Uncaria rhynchophylla (Miq.) Miq. ex Havil. / related accepted Uncaria spp., Gastrodia elata Blume, Ligusticum chuanxiong Hort., Angelica dahurica (Hoffm.) Benth. & Hook.f. ex Franch. & Sav., Tribulus terrestris L., Angelica sinensis (Oliv.) Diels, Polygonum multiflorum Thunb. [stem drug], Rehmannia glutinosa (Gaertn.) DC., Paeonia lactiflora Pall., and Glycyrrhiza spp.; mineral drugs also reported (Haematitum) | One dose daily; decocted twice; 150 mL each time, morning and evening; treatment for 4 weeks | 2 = Limited |
| Zhang Qiuxiang_2019 | Yangxue Pinggan Decoction | Not identified in the study | Described as an empirical formula of Professor Guan Youbo | Inulae Flos; Haematitum; Gypsum Fibrosum; Angelicae Sinensis Radix; Chuanxiong Rhizoma; Rehmanniae Radix; Paeoniae Radix Alba; Polygoni Multiflori Caulis; Cyperi Rhizoma; Glycyrrhizae Radix et Rhizoma | Inula spp. [for Inulae Flos], Angelica sinensis (Oliv.) Diels, Ligusticum chuanxiong Hort., Rehmannia glutinosa (Gaertn.) DC., Paeonia lactiflora Pall., Polygonum multiflorum Thunb. [stem drug], Cyperus rotundus L., and Glycyrrhiza spp.; mineral drugs also reported (Haematitum, Gypsum Fibrosum) | Continuous administration for 4 weeks; the currently retrievable uploaded excerpt does not preserve the full dose line | 2 = Limited |
| Wang Yanfeng_2019 | Yangxue Pinggan Decoction | Not identified in the study | Not stated as a formal pharmacopoeial/regulatory standard; the article presents a study-specific decoction for migraine | Margaritae Concha 30 g; Haliotidis Concha 30 g; Uncariae Ramulus cum Uncis 20 g; Gastrodiae Rhizoma 12 g; Chuanxiong Rhizoma 12 g; Angelicae Dahuricae Radix 12 g; Paeoniae Radix Alba 10 g; Tribuli Fructus 10 g; Angelicae Sinensis Radix 10 g; Rehmanniae Radix 10 g | Pteria martensii (Dunker), Haliotis spp., Uncaria rhynchophylla (Miq.) Miq. ex Havil. / related accepted Uncaria spp., Gastrodia elata Blume, Ligusticum chuanxiong Hort., Angelica dahurica (Hoffm.) Benth. & Hook.f. ex Franch. & Sav., Paeonia lactiflora Pall., Tribulus terrestris L., Angelica sinensis (Oliv.) Diels, and Rehmannia glutinosa (Gaertn. | Water-decocted; 1 dose/day; taken twice daily morning and evening; combined with oral flunarizine; treatment for 2 months | 2 = Limited |
| Li Dongyan_2020 | Banxia Baizhu Tianma Decoction | Not identified in the study | Not stated | Not stated | Not assessable | Western medicine plus Banxia Baizhu Tianma Decoction in the treatment group; exact composition and preparation details not retrievable from the current uploaded text | 3 = Inadequate |
| Yang Fan_2022 | Banxia Baizhu Tianma Decoction | Not identified in the study | Not stated | Not stated | Not assessable | Oral modified Banxia Baizhu Tianma Decoction added to conventional treatment; used in acute attack over the study treatment period; exact composition and preparation details not retrievable from current text | 3 = Inadequate |
| Ou Jianghong_2016 | Banxia Baizhu Tianma Decoction | Not identified in the study | Not stated | Not stated | Not assessable | Observation group received modified Banxia Baizhu Tianma Decoction for 6 weeks; exact ingredient list and preparation details not retrievable from current text | 3 = Inadequate |
| Zheng Quancheng_2021 | Banxia Baizhu Tianma Decoction | Not identified in the study | The article reports modified Banxia Baizhu Tianma Decoction combined with acupuncture for migraine with wind-phlegm upset syndrome | Not stated | Not assessable | Oral decoction at 1 dose/day combined with acupuncture and placebo-controlled comparator; treatment for 4 weeks; full formula composition not recoverable from the currently retrievable uploaded text | 3 = Inadequate |
| Guo Xiaoyun_2013 | Banxia Baizhu Tianma Decoction | Not identified in the study | The article reports modified Banxia Baizhu Tianma Decoction for migraine with phlegm-turbidity disturbing upward syndrome | Not stated | Not assessable | Treatment course 15 days; detailed preparation/composition not recoverable from the currently retrievable uploaded text | 3 = Inadequate |
| Chen Shaomei_2003 | Banxia Baizhu Tianma Decoction | Not identified in the study | The title of the uploaded article identifies Sanchong Banxia Baizhu Tianma Decoction for migraine, but the garbled text does not allow reliable extraction of formula provenance | Not stated | Not assessable | Not stated | 3 = Inadequate |
| HW Liu_2018 | Wuzhuyu Decoction | Not identified in the study | Not identifiable from the uploaded excerpt; the article reports a modified formula for migraine with cold and stasis obstructing meridian syndrome | Not stated | Not assessable | Granule formulation: treatment course lasted 12 weeks with 4-week follow-up; detailed preparation/composition not available in the retrieved uploaded text | 3 = Inadequate |
| Liu Hongyan_2006 | Wuzhuyu Decoction | Not identified in the study | Not identifiable from the uploaded copy | Not extractable | Not assessable | Not extractable | 3 = Inadequate |
| Zhang Honglin_2024 | Wuzhuyu Decoction | classical Wuzhuyu Decoction from Shanghan Zabing Lun | Linked to classical Wuzhuyu Decoction in Shanghan Zabing Lun | Evodiae Fructus 1 sheng; Ginseng 3 liang; Jujubae Fructus 12 pieces; Zingiberis Rhizoma Recens 6 liang; with symptom-based additions including Xiakucao, Heye, Huangqin, Juhua, Huainiuxi, and others depending on presentation | Tetradium ruticarpum (A.Juss.) T.G.Hartley [for Evodiae Fructus], Panax ginseng C.A.Mey., Ziziphus jujuba Mill., and Zingiber officinale Roscoe; additional modified ingredients included Prunella vulgaris L., Nelumbo nucifera Gaertn., Scutellaria baicalensis Georgi, Chrysanthemum × morifolium (Ramat.) | Water seven sheng, decocted to two sheng, dregs removed, warm administration in divided doses; symptom-based additions described in text; detailed modern gram standardization not consistently provided | 2 = Limited |
| Li Zhijin_2019 | Wuzhuyu Decoction | Not identified in the study | Linked to classical Wuzhuyu Decoction in the article title and narrative, but the currently retrievable uploaded text does not preserve the methods-level ingredient list | Not stated | Not assessable | Oral Wuzhuyu Decoction combined with warm acupuncture; treatment course 8 weeks with 4-week follow-up | 3 = Inadequate |
| Liu Hongwei_2022 | Wuzhuyu Decoction | Wuzhuyu Decoction to Shanghan Lun | Related to classical Wuzhuyu Decoction from Shanghan Lun; the study used a modified formula | Evodiae Fructus 6 g; Chuanxiong Rhizoma 12 g; Angelicae Dahuricae Radix 12 g; Menthae Herba 6 g | Tetradium ruticarpum (A.Juss.) T.G.Hartley [for Evodiae Fructus], Ligusticum chuanxiong Hort., Angelica dahurica (Hoffm.) Benth. & Hook.f. ex Franch. & Sav., and Mentha canadensis L. / related accepted medicinal Mentha taxon [for Menthae Herba] | Granules from Guangdong Yifang Pharmaceutical; dissolved in boiling water to 200 mL; taken morning and evening; once daily; treatment for 12 weeks | 2 = Limited |
| Ge Xuehua_2023 | Sanpian Decoction | Not identified in the study | Not stated | Salviae Miltiorrhizae Radix et Rhizoma 30 g; Uncariae Ramulus cum Uncis 30 g; Achyranthis Bidentatae Radix 30 g; Chuanxiong Rhizoma 30 g; Viticis Fructus 30 g; Corydalis Rhizoma 15 g; Eupolyphaga 10 g; Angelicae Dahuricae Radix 6 g; Glycyrrhizae Radix et Rhizoma 6 g; Asarum 3 g; Scorpio 3 g | Salvia miltiorrhiza Bunge, Uncaria rhynchophylla (Miq.) Miq. ex Havil. / related accepted Uncaria spp., Achyranthes bidentata Blume, Ligusticum chuanxiong Hort., Vitex trifolia L. var. simplicifolia Cham. / accepted source for Viticis Fructus, Corydalis yanhusuo W.T.Wang, Eupolyphaga sinensis Walker, Angelica dahurica (Hoffm.) | Soaked with 500 mL water for 30 min; decocted over gentle fire for about 20–25 min; decocted twice; mixed; taken warm twice daily; treated for more than 3 months | 2 = Limited |
| Li Lili_2025 | Sanpian Decoction | Not identified in the study | Not stated | Not stated | Not assessable | Oral modified Sanpian decoction granules for 4 weeks; placebo granules used in control group; rescue ibuprofen allowed for intolerable headache | 3 = Inadequate |
| Li Ruijie_2013 | Sanpian Decoction | Not identified in the study | Not stated | Chuanxiong Rhizoma 15 g; Paeoniae Radix Alba 10 g; Bupleuri Radix 10 g; Cyperi Rhizoma 10 g; Angelicae Dahuricae Radix 12 g; Pheretima 10 g; Ligustici Rhizoma 9 g; Menthae Herba 6 g; remaining ingredients not preserved in the currently retrievable uploaded text | Ligusticum chuanxiong Hort., Paeonia lactiflora Pall., Bupleurum chinense DC. / B. scorzonerifolium Willd., Cyperus rotundus L., Angelica dahurica (Hoffm.) Benth. & Hook.f. ex Franch. & Sav., Pheretima aspergillum (E. Perrier) / related accepted earthworm source [for Pheretima], Ligusticum sinense Oliv. or related accepted source [for Ligustici Rhizoma] | Oral modified Sanpian Decoction for 3 months; exact full preparation details not fully preserved in the retrievable uploaded text | 2 = Limited |
| Li Hui_2012 | Sanpian Decoction | Not identified in the study | Not stated | Chuanxiong Rhizoma 15 g; Paeoniae Radix Alba 10 g; Bupleuri Radix 10 g; Cyperi Rhizoma 10 g; Angelicae Dahuricae Radix 12 g; Pheretima 10 g; Ligustici Rhizoma 9 g; Menthae Herba 6 g; remaining ingredients not preserved in the currently retrievable uploaded text | Ligusticum chuanxiong Hort., Paeonia lactiflora Pall., Bupleurum chinense DC. / B. scorzonerifolium Willd., Cyperus rotundus L., Angelica dahurica (Hoffm.) Benth. & Hook.f. ex Franch. & Sav., Pheretima spp., Ligusticum spp | Oral modified Sanpian Decoction for 1 month; exact full preparation details not fully preserved in the retrievable uploaded text | 2 = Limited |
| Liang Zengkun_2011 | Sanpian Decoction | Not identified in the study | Not stated | Not stated | Not assessable | 1 dose/day after water decoction, divided morning and evening; combined with daily electroacupuncture for 30 min; both groups treated continuously for 1 month | 3 = Inadequate |
| Shen Bin_2016 | Sanpian Decoction | Not identified in the study | The article reports Chinese medicine granules of modified Sanpian Decoction for migraine of liver stagnation and blood stasis syndrome | Not stated | Not assessable | Granule formulation; treatment for 4 weeks with follow-up 4 weeks later; detailed formula composition was not recoverable from the currently retrievable uploaded text | 3 = Inadequate |
| Gu Hongbing_2023 | Sanpian Decoction | Not identified in the study | The article reports Sanpian Decoction combined with acupuncture for migraine | Not stated | Not assessable | Combined with acupuncture; treatment for 4 weeks; full herb composition and preparation were not recoverable from the currently retrievable uploaded text | 3 = Inadequate |
| Ma Kaixuan_2025 | Sanpian Decoction | Not identified in the study | The article states that Sanpian Decoction originates from Bianzheng Lu and is used for shaoyang headache caused by external wind obstructing the surface and impairing qi movement | Chuanxiong Rhizoma 20 g; Angelicae Sinensis Radix 15 g; Angelicae Dahuricae Radix 15 g; Schizonepetae Spica 12 g; Cinnamomi Ramulus 12 g; Cyperi Rhizoma 12 g; Bupleuri Radix 12 g; Paeoniae Radix Alba 12 g; Glycyrrhizae Radix et Rhizoma 6 g | Ligusticum chuanxiong Hort., Angelica sinensis (Oliv.) Diels, Angelica dahurica (Hoffm.) Benth. & Hook.f. ex Franch. & Sav., Schizonepeta tenuifolia Briq., Cinnamomum cassia (L.) | Decocted in hospital pharmacy; water-decocted to obtain 200 mL; taken twice daily; continuous treatment for 4 weeks | 2 = Limited |
| Wan Huiqin_2014 | Xuefu Zhuyu Decoction | Not identified in the study | Not stated in the retrieved uploaded text | Chuanxiong Rhizoma 20 g; Angelicae Sinensis Radix 15 g; Angelicae Dahuricae Radix 15 g; Schizonepetae Spica 12 g; Cinnamomi Ramulus 12 g; Cyperi Rhizoma 12 g; Bupleuri Radix 12 g; Paeoniae Radix Alba 12 g; Glycyrrhizae Radix et Rhizoma 6 g | Not assessable | Not stated | 3 = Inadequate |
| Liu Yuhong_2015 | Xuefu Zhuyu Decoction | Not identified in the study | Not stated in the retrieved uploaded text | Chuanxiong Rhizoma 20 g; Angelicae Sinensis Radix 15 g; Angelicae Dahuricae Radix 15 g; Schizonepetae Spica 12 g; Cinnamomi Ramulus 12 g; Cyperi Rhizoma 12 g; Bupleuri Radix 12 g; Paeoniae Radix Alba 12 g; Glycyrrhizae Radix et Rhizoma 6 g | Not assessable | Not stated | 3 = Inadequate |
| Liu Fei_2018 | Xuefu Zhuyu Decoction | Not identified in the study | Based on Xuefu Zhuyu Decoction | Persicae Semen 15 g; Carthami Flos 15 g; Achyranthis Bidentatae Radix 12 g; Paeoniae Radix Rubra 12 g; Bupleuri Radix 12 g; Platycodonis Radix 9 g; Aurantii Fructus 9 g; Angelicae Sinensis Radix 9 g; Rehmanniae Radix 9 g; Chuanxiong Rhizoma 9 g; Glycyrrhizae Radix et Rhizoma 6 g; optional additions: Ephedra, processed Chuanwu, and Cinnamomi Ramulus for wind-cold; Atractylodes and Pericarpium Citri for wind-damp; Bambusae Caulis in Taeniam and Arisaema cum Bile for phlegm turbidity; Astragalus and Codonopsis for qi-blood deficiency | Prunus persica (L.) Batsch [for Persicae Semen], Carthamus tinctorius L., Achyranthes bidentata Blume, Paeonia veitchii Lynch / accepted source for Paeoniae Radix Rubra, Bupleurum chinense DC. / B. scorzonerifolium Willd., Platycodon grandiflorus (Jacq.) A.DC., Citrus × aurantium L. [for Aurantii Fructus], Angelica sinensis (Oliv.) | One dose daily; water-decocted and divided for administration; continuous treatment for 30 days with a 5-days-on/2-days-off cycle | 2 = Limited |
| Shi Hongyan_2023 | Xuefu Zhuyu Decoction | Xuefu Zhuyu Decoction originates from Yilin Gaicuo | Origin explicitly attributed to Wang Qingren's Yilin Gaicuo | Angelicae Sinensis Radix 15 g; Achyranthis Bidentatae Radix 15 g; Persicae Semen 12 g; Carthami Flos 12 g; Rehmanniae Radix 12 g; bran-fried Aurantii Fructus 12 g; Corydalis Rhizoma 12 g; Chuanxiong Rhizoma 9 g; Paeoniae Radix Rubra 9 g; Platycodonis Radix 9 g; Bupleuri Radix 9 g; Ophiopogonis Radix 9 g; honey-fried Glycyrrhizae Radix et Rhizoma 6 g; optional additions for qi stagnation, qi deficiency, and severe pain | Angelica sinensis (Oliv.) Diels, Achyranthes bidentata Blume, Prunus persica (L.) Batsch, Carthamus tinctorius L., Rehmannia glutinosa (Gaertn.) DC., Citrus × aurantium L., Corydalis yanhusuo W.T.Wang, Ligusticum chuanxiong Hort., Paeonia veitchii Lynch / accepted source for Paeoniae Radix Rubra, Platycodon grandiflorus (Jacq.) | Prepared by the hospital decoction room and decocted to 400 mL; administered as 200 mL twice daily after meals; treatment duration 45 days | 2 = Limited |
| Zhang Bo_2011 | Xuefu Zhuyu Decoction | Not identified in the study | The discussion identifies Xuefu Zhuyu Decoction as deriving from Yilin Gaicuo | The uploaded paper explicitly identifies the base formula structure as Tao Hong Siwu Tang plus Sini San with Jiegeng and Niuxi; it describes the constituent herbs as Danggui, Chuanxiong, Taoren, Chishao, Honghua, Niuxi, Chaihu, Jiegeng, Zhike, Shengdi, and Gancao, but the current retrievable text does not preserve a single clean methods-level dose line for all herbs | Angelica sinensis (Oliv.) Diels, Rehmannia glutinosa (Gaertn.) DC., Prunus persica (L.) Batsch, Carthamus tinctorius L., Achyranthes bidentata Blume, Bupleurum chinense DC. / B. scorzonerifolium Willd., Platycodon grandiflorus (Jacq.) | Daily water-decocted administration; the uploaded copy indicates 200 mL per day divided morning/evening and one-month treatment in the parsed page | 2 = Limited |
| Cao Li_2015 | Xuefu Zhuyu Decoction | Not identified in the study | Not stated | Angelicae Sinensis Radix 20 g; Chuanxiong Rhizoma 20 g; Paeoniae Radix Rubra 15 g; Carthami Flos 15 g; Persicae Semen 15 g; Achyranthis Bidentatae Radix 12 g; Platycodonis Radix 12 g; Aurantii Fructus 12 g; Bupleuri Radix 10 g; Glycyrrhizae Radix et Rhizoma 6 g; combined with Tongxinluo capsules | Angelica sinensis (Oliv.) Diels, Ligusticum chuanxiong Hort., Paeonia veitchii Lynch / accepted source for Paeoniae Radix Rubra, Carthamus tinctorius L., Prunus persica (L.) Batsch, Achyranthes bidentata Blume, Platycodon grandiflorus (Jacq.) | Xuefu Zhuyu Decoction water-decocted, 1 dose/day, administered three times daily, 100 mL each time; 2 weeks per course for 2 courses | 2 = Limited |
| Li Bin_2018 | Xuefu Zhuyu Decoction | Not identified in the study | Not stated | Not stated | Not assessable | Combined modified Xuefu Zhuyu Decoction with western medicine; preparation details not retrievable from the current uploaded text | 3 = Inadequate |
| Li Zhishen_2025 | Xuefu Zhuyu Decoction | Not identified in the study | The discussion identifies Xuefu Zhuyu Decoction as a classical blood-stasis formula, but no formal regulatory standard is cited in the retrievable excerpt | Not stated | Not assessable | Cyproheptadine hydrochloride plus Xuefu Zhuyu Decoction for 4 weeks; exact ingredient list and decoction procedure not retrievable from the current uploaded text | 3 = Inadequate |
| Wang Ping_2016 | Xuefu Zhuyu Decoction | Not identified in the study | The article explicitly attributes Xuefu Zhuyu Decoction to Wang Qingren | Angelicae Sinensis Radix 12 g; Rehmanniae Radix 12 g; Persicae Semen 10 g; Carthami Flos 10 g; Aurantii Fructus 10 g; Paeoniae Radix Rubra 10 g; Bupleuri Radix 10 g; Glycyrrhizae Radix et Rhizoma 6 g; Platycodonis Radix 6 g; Chuanxiong Rhizoma 6 g; Achyranthis Bidentatae Radix 6 g; Astragali Radix 15 g; Uncariae Ramulus cum Uncis 15 g; with syndrome-based additions including Asarum, Notopterygium, Pinellia, Gastrodia, Haliotidis Concha, Chrysanthemum, Salvia, Pheretima, Moutan Cortex, Gardenia, Shenjincao, Sigualuo, Scorpio, Notoginseng powder, Rehmanniae Radix Preparata, Ejiao, Ziziphi Spinosae Semen, and Rhei Radix et Rhizoma | Angelica sinensis (Oliv.) Diels, Rehmannia glutinosa (Gaertn.) DC., Prunus persica (L.) Batsch, Carthamus tinctorius L., Citrus × aurantium L., Paeonia veitchii Lynch / accepted source for Paeoniae Radix Rubra, Bupleurum chinense DC. / B. scorzonerifolium Willd., Glycyrrhiza spp., Platycodon grandiflorus (Jacq.) | Water-decocted to 400 mL; 1 dose/day; taken warm twice daily; combined with oral nimodipine; treatment for 4 courses as described in the article | 2 = Limited |
| Bai Yuexiu_2023 | Xuefu Zhuyu Decoction | Not identified in the study | The article states that the treatment group received Xuefu Zhuyu Decoction combined with modified Shengjiang Powder on top of basic western medicine | Not stated | Not assessable | Combined with basic western medicine; treatment duration and detailed administration beyond the study summary were not recoverable from the currently retrievable uploaded text | 3 = Inadequate |
| Huo Lirong_2017 | Xuefu Zhuyu Decoction | Not identified in the study | The article presents modified Xuefu Zhuyu Decoction for acute migraine without citing a formal pharmacopoeial/regulatory standard | Angelicae Sinensis Radix 10 g; Mori Folium 10 g; Glycyrrhizae Radix et Rhizoma 6 g; Rehmanniae Radix 15 g; Platycodonis Radix 5 g; Persicae Semen 10 g; Carthami Flos 5 g; Aurantii Fructus 10 g; Chuanxiong Rhizoma 20 g; Achyranthis Bidentatae Radix 20 g; Paeoniae Radix Rubra and Paeoniae Radix Alba 20 g each; Scolopendra 1 piece; with syndrome-based additions as reported in the article | Angelica sinensis (Oliv.) Diels, Morus alba L. [for Mori Folium], Glycyrrhiza spp., Rehmannia glutinosa (Gaertn.) DC., Platycodon grandiflorus (Jacq.) A.DC., Prunus persica (L.) Batsch, Carthamus tinctorius L., Citrus × aurantium L., Ligusticum chuanxiong Hort., Achyranthes bidentata Blume, Paeonia lactiflora Pall. | Oral decoction; compared against nimodipine and oryzanol; exact decoction volume was not fully retrievable from the currently available text | 2 = Limited |
| Ma Yongsheng_2016 | Xuefu Zhuyu Decoction | Not identified in the study | Not stated in the article; the prescription was used as a study formula | Bupleuri Radix 10 g; Paeoniae Radix Rubra 20 g; Aurantii Fructus 15 g; Persicae Semen 15 g; Carthami Flos 15 g; Rehmanniae Radix 30 g; Chuanxiong Rhizoma 15 g; Angelicae Sinensis Radix 30 g; Achyranthis Bidentatae Radix 30 g; Platycodonis Radix 10 g; Scorpio 5 g; Glycyrrhizae Radix et Rhizoma 5 g; syndrome-based additions: Arisaema cum Bile 10 g, Pinelliae Rhizoma Praeparatum 15 g, Acori Tatarinowii Rhizoma 30 g for phlegm-stasis obstruction; Haliotidis Concha, Uncariae Ramulus cum Uncis, Gastrodiae Rhizoma 10 g each for yin deficiency and yang hyperactivity; Codonopsis Radix and Astragali Radix 30 g each for qi deficiency and blood stasis | Bupleurum chinense DC. / B. scorzonerifolium Willd., Paeonia veitchii Lynch, Citrus × aurantium L., Prunus persica (L.) Batsch, Carthamus tinctorius L., Rehmannia glutinosa (Gaertn.) DC., Ligusticum chuanxiong Hort., Angelica sinensis (Oliv.) | Water-decocted to 300 mL; 1 dose/day, divided morning and evening; combined with flunarizine 5 mg twice daily and fluoxetine 20 mg once daily; treatment for 2 weeks | 2 = Limited |
| Wei Ping_2015 | Xuefu Zhuyu Decoction | Not identified in the study | The article describes the formula as based on Xuefu Zhuyu Decoction | Angelicae Sinensis Radix 9 g; Rehmanniae Radix 9 g; Persicae Semen 12 g; Carthami Flos 3 g; Aurantii Fructus 6 g; Paeoniae Radix Rubra 6 g; Bupleuri Radix 3 g; Glycyrrhizae Radix et Rhizoma 6 g; Platycodonis Radix 5 g; Chuanxiong Rhizoma 5 g; Achyranthis Bidentatae Radix 9 g; syndrome-based additions: Ephedra, Cinnamomi Ramulus, and processed Aconiti Radix for severe wind-cold; Lonicerae Flos and Gardeniae Fructus for wind-heat; Atractylodis Rhizoma and Citri Pericarpium for wind-dampness; Bambusae Caulis in Taeniam and Arisaema cum Bile for phlegm turbidity; Gastrodiae Rhizoma and Uncariae Ramulus cum Uncis for liver-yang hyperactivity; Astragali Radix and Codonopsis Radix for qi-blood deficiency | Angelica sinensis (Oliv.) Diels, Rehmannia glutinosa (Gaertn.) DC., Prunus persica (L.) Batsch, Carthamus tinctorius L., Citrus × aurantium L., Paeonia veitchii Lynch, Bupleurum chinense DC. / B. scorzonerifolium Willd., Glycyrrhiza spp., Platycodon grandiflorus (Jacq.) A.DC., Ligusticum chuanxiong Hort., and Achyranthes bidentata Blume, with syndrome-based additions including Ephedra sinica Stapf, Cinnamomum cassia (L.) J.Presl, Lonicera japonica Thunb., Gardenia jasminoides J.Ellis, Atractylodes lancea (Thunb.) | 1 dose/day, water-decocted and divided for oral administration; continuous medication for 5 days per course, 4 courses in total; flunarizine 10 mg once daily at bedtime in both groups | 2 = Limited |
| Li Weixiong_2016 | Xuefu Zhuyu Decoction | Not identified in the study | Not stated in the article | Bupleuri Radix 10 g; Paeoniae Radix Rubra 20 g; Aurantii Fructus 15 g; Persicae Semen 15 g; Carthami Flos 15 g; Rehmanniae Radix 30 g; Chuanxiong Rhizoma 15 g; Angelicae Sinensis Radix 30 g; Achyranthis Bidentatae Radix 30 g; Platycodonis Radix 10 g; Scorpio 5 g; Glycyrrhizae Radix et Rhizoma 5 g; syndrome-based additions: Arisaema cum Bile 10 g, Pinelliae Rhizoma Praeparatum 15 g, Acori Tatarinowii Rhizoma 30 g for phlegm-stasis obstruction; Haliotidis Concha, Uncariae Ramulus cum Uncis, Gastrodiae Rhizoma 10 g each for yin deficiency and yang hyperactivity; Codonopsis Radix and Astragali Radix 30 g each for qi deficiency and blood stasis | Bupleurum chinense DC. / B. scorzonerifolium Willd., Paeonia veitchii Lynch, Citrus × aurantium L., Prunus persica (L.) Batsch, Carthamus tinctorius L., Rehmannia glutinosa (Gaertn.) DC., Ligusticum chuanxiong Hort., Angelica sinensis (Oliv.) Diels, Achyranthes bidentata Blume, Platycodon grandiflorus (Jacq.) | Water-decocted to 300 mL; 1 dose/day, divided morning and evening; combined with flunarizine 5 mg twice daily and fluoxetine 20 mg once daily; treatment for 2 weeks | 2 = Limited |
| Li Tuanhui_2016 | Xuefu Zhuyu Decoction | Not identified in the study | Not stated in the article | Chuanxiong Rhizoma 15 g; Persicae Semen 15 g; Rehmanniae Radix 15 g; Achyranthis Bidentatae Radix 15 g; Paeoniae Radix Rubra 15 g; Angelicae Sinensis Radix 15 g; Carthami Flos 10 g; Platycodonis Radix 10 g; Bupleuri Radix 6 g; Glycyrrhizae Radix et Rhizoma 3 g; Aurantii Fructus 6 g; syndrome-based additions: Rehmanniae Radix Praeparata 30 g, Radix Pseudostellariae 20 g, Corni Fructus 15 g, Eucommiae Cortex 20 g, Lycii Fructus 30 g for kidney deficiency; Atractylodis Macrocephalae Rhizoma 15 g, Citri Pericarpium 15 g, Pinelliae Rhizoma 15 g, Gastrodiae Rhizoma 10 g, Scutellariae Radix 10 g for phlegm turbidity; Inulae Flos 10 g, Bambusae Caulis in Taeniam 10 g, Haematitum 10 g for nausea and vomiting; Corydalis Rhizoma 10 g, Curcumae Radix 10 g, 2 centipedes, Scorpio 6 g for blood stasis; Gastrodiae Rhizoma 10 g, Haliotidis Concha 15 g, Gardeniae Fructus 15 g, Uncariae Ramulus cum Uncis 15 g, Tribuli Fructus 15 g for liver-yang pattern | Ligusticum chuanxiong Hort., Prunus persica (L.) Batsch, Rehmannia glutinosa (Gaertn.) DC., Achyranthes bidentata Blume, Paeonia veitchii Lynch, Angelica sinensis (Oliv.) Diels, Carthamus tinctorius L., Platycodon grandiflorus (Jacq.) A.DC., Bupleurum chinense DC. / B. scorzonerifolium Willd., Glycyrrhiza spp., and Citrus × aurantium L., with multiple syndrome-based additions including Cornus officinalis Siebold & Zucc., Eucommia ulmoides Oliv., Lycium barbarum L., Pinellia ternata (Thunb.) | 1 dose/day, water-decocted and taken in two divided doses morning and evening; combined with flunarizine 10 mg once daily at bedtime; continuous medication for 8 days | 2 = Limited |
| Ye Sheng_2023 | Tongqiao Huoxue Decoction | Not identified in the study | Discussed as a traditional formula; specific authoritative source not recoverable from the current uploaded snippet | The current retrievable uploaded text only confirms core herbs discussed in the paper narrative, including Persicae Semen, Paeoniae Radix Rubra, Carthami Flos, Chuanxiong Rhizoma, Achyranthis/Niuxi, and Scorpio/Quanxie; a full clean methods-level composition with all doses is not retrievable from the current uploaded text | Prunus persica (L.) Batsch, Paeonia veitchii Lynch, Carthamus tinctorius L., Ligusticum chuanxiong Hort., Achyranthes bidentata Blume / related source for Niuxi, and Buthus martensii Karsch | Not fully extractable from the currently retrievable uploaded text | 3 = Inadequate |
| Zhang Qingli_2021 | Tongqiao Huoxue Decoction | Not identified in the study | Not clearly stated in the retrieved uploaded excerpt | Persicae Semen 10 g; Carthami Flos 10 g; Chuanxiong Rhizoma 15 g; Paeoniae Radix Rubra 15 g; Pheretima 10 g; Angelicae Sinensis Radix 15 g; wine-processed Rhei Radix et Rhizoma 10 g; Cyperi Rhizoma 15 g; Bupleuri Radix 15 g; Hordei Fructus Germinatus 30 g; Cyathulae Radix 15 g; Scorpio 5 g | Prunus persica (L.) Batsch, Carthamus tinctorius L., Ligusticum chuanxiong Hort., Paeonia veitchii Lynch, Pheretima spp., Angelica sinensis (Oliv.) Diels, Rheum palmatum L. / related accepted source [for Rhei Radix et Rhizoma] | One dose daily; water-decocted and taken warm in two divided doses; treatment for 3 weeks | 2 = Limited |
| Li Jing_2016 | Tongqiao Huoxue Decoction | Not identified in the study | Not stated | Not fully extractable from the currently retrievable uploaded text; visible dose fragments include 20 g, 10 g, 5 g, and 3 g, but the ingredient labels are not reliably preserved | Not assessable | Tongqiao Huoxue Decoction combined with flunarizine; prepared to 200 mL; treatment and observation recorded over 14 days | 3 = Inadequate |
| Liang Shucheng_2021 | Tongqiao Huoxue Decoction | Not identified in the study | Not stated | Not stated | Not assessable | Tongqiao Huoxue Decoction combined with wrist-ankle acupuncture on top of conventional western medicine; exact composition and preparation details not retrievable from the current uploaded text | 3 = Inadequate |
| Wang Ying_2017 | Tongqiao Huoxue Decoction | Not identified in the study | The article describes Tongqiao Huoxue Decoction as the treatment formula for blood-stasis migraine, but does not cite a formal pharmacopoeial/regulatory standard | Chuanxiong Rhizoma 20 g; Paeoniae Radix Rubra 20 g; Carthami Flos 20 g; Persicae Semen 20 g; Allii Fistulosi Bulbus 3 g (chopped old scallion); Jujubae Fructus 7 pieces; Moschus 0.05 g (taken separately); with syndrome-based additions including Scolopendra, Eupolyphaga/ground beetle material, Scorpio, Astragali Radix, Radix Pseudostellariae, Polygoni Multiflori Caulis/Polygonum multiflorum, Polygonatum, Colla Corii Asini, Pinelliae Rhizoma, Poria, Albizia flower, Acorus, and Polygoni Multiflori Caulis/Night-intertwining vine as reported | Ligusticum chuanxiong Hort., Paeonia veitchii Lynch, Carthamus tinctorius L., Prunus persica (L.) Batsch, Allium fistulosum L., Ziziphus jujuba Mill., and Moschus [animal drug; exact zoological source not standardizable from the report] | Water-decocted to 400 mL; taken orally morning and evening; combined with flunarizine 5 mg twice daily; 28 days per course with 3-month follow-up | 2 = Limited |
| Su Chengcai_2015 | Tongqiao Huoxue Decoction | Not identified in the study | The article presents Tongqiao Huoxue Decoction as a traditional formula for migraine, without citing a formal pharmacopoeial/regulatory standard | Zingiberis Rhizoma Recens 9 g; Persicae Semen 10 g; Carthami Flos 10 g; Allii Fistulosi Bulbus 12 g; Jujubae Fructus 15 g; Paeoniae Radix Rubra 15 g; Chuanxiong Rhizoma 15 g; Angelicae Dahuricae Radix 30 g; with syndrome-based additions including Gardeniae Fructus 9 g and Tribuli Fructus 15 g for liver-fire exuberance, and additional unspecified modifications reported in the article | Zingiber officinale Roscoe, Prunus persica (L.) Batsch, Carthamus tinctorius L., Allium fistulosum L., Ziziphus jujuba Mill., Paeonia veitchii Lynch, Ligusticum chuanxiong Hort., and Angelica dahurica (Hoffm.) | Continuous treatment for 3 months; oral decoction administration; exact daily decoction volume was not fully recoverable from the currently retrievable uploaded text | 2 = Limited |
| Zhao Yunxia_2023 | Tongqiao Huoxue Decoction | Not identified in the study | The article presents modified Tongqiao Huoxue Decoction for migraine without citing a formal pharmacopoeial/regulatory standard | Persicae Semen 15 g; Angelicae Sinensis Radix 15 g; Chuanxiong Rhizoma 10 g; Angelicae Dahuricae Radix 10 g; Paeoniae Radix Rubra 10 g; Carthami Flos 5 g; Allii Fistulosi Bulbus 5 g; Zingiberis Rhizoma Recens 5 g; with additions of Citri Reticulatae Pericarpium 10 g and Pinelliae Rhizoma 10 g for nausea/vomiting, Gastrodiae Rhizoma 10 g and Uncariae Ramulus cum Uncis 10 g for vertigo, and Asarum 10 g plus Cinnamomi Ramulus 8 g when induced or aggravated by cold | Prunus persica (L.) Batsch, Angelica sinensis (Oliv.) Diels, Ligusticum chuanxiong Hort., Angelica dahurica (Hoffm.) Benth. & Hook.f. ex Franch. & Sav., Paeonia veitchii Lynch, Carthamus tinctorius L., Allium fistulosum L., Zingiber officinale Roscoe, Citrus reticulata Blanco, Pinellia ternata (Thunb.) | Water-decocted; 1 dose/day; 400 mL divided into morning and evening doses; combined with daily warm acupuncture; treatment for 4 weeks | 2 = Limited |
| Guo Jinqiao_2023 | Tongqiao Huoxue Decoction | Not identified in the study | The article reports modified Tongqiao Huoxue Decoction combined with flunarizine for vestibular migraine | Not stated | Not assessable | Combined with flunarizine; treatment details other than the study summary were not recoverable from the currently retrievable uploaded text | 3 = Inadequate |
| Qian Yuliang_2006 | Tongqiao Huoxue Decoction | Not identified in the study | The article presents Tongqiao Huoxue Decoction as a therapeutic formula for migraine, without citing a formal pharmacopoeial/regulatory standard | Paeoniae Radix Rubra 15 g; Chuanxiong Rhizoma 15 g; Persicae Semen 10 g; Carthami Flos 10 g; Angelicae Dahuricae Radix 30 g; Allii Fistulosi Bulbus 12 g; Zingiberis Rhizoma Recens 9 g; Jujubae Fructus 15 g; yellow rice wine 100 mL (taken with the decoction); Salviae Miltiorrhizae Radix et Rhizoma 30 g; Astragali Radix 20 g; with additions including Gardeniae Fructus 9 g and Tribuli Fructus 15 g for exuberant liver fire, Bombyx Batryticatus 12 g and Scolopendra 2 pieces for severe radiating pain, Pinelliae Rhizoma 9 g and Bambusae Caulis in Taeniam 12 g for phlegm turbidity, Puerariae Lobatae Radix and Gastrodiae Rhizoma for frontal headache, Chrysanthemi Flos and Viticis Fructus for bilateral headache, Evodiae Fructus for vertex headache, and Inulae Flos plus Bambusae Caulis in Taeniam for nausea/vomiting | Not assessable | 1 dose/day; decocted to 250 mL; taken in 2-3 divided doses; treatment for 28 days | 2 = Limited |
| Chen Shanshan_2014 | Tongqiao Huoxue Decoction | Not identified in the study | The uploaded file appears to concern coronary heart disease angina rather than migraine herbal composition reporting | Not applicable | Not assessable | Not applicable | 3 = Inadequate |
| Gao Junning_2019 | Tongqiao Huoxue Decoction | Not identified in the study | Not clearly stated in the article | Astragali Radix Praeparata 30 g; Carthami Flos 15 g; Allii Fistulosi Bulbus 15 g; Persicae Semen 10 g; Paeoniae Radix Rubra 12 g; Chuanxiong Rhizoma 12 g; Ligustici Rhizoma 10 g; Angelicae Dahuricae Radix 10 g; Glycyrrhizae Radix et Rhizoma 6 g; Scorpio 6 g; Bombyx Batryticatus 6 g; Asarum 3 g; syndrome-based additions included Saposhnikoviae Radix and Notopterygii Rhizoma for taiyang headache, Puerariae Lobatae Radix and Viticis Fructus for yangming headache, Bupleuri Radix and Scutellariae Radix for shaoyang headache, Pinelliae Rhizoma and Atractylodis Rhizoma for taiyin headache, Asarum 6-9 g for shaoyin headache, Ligustici Rhizoma and Evodiae Fructus for jueyin headache, Gastrodiae Rhizoma and Haliotidis Concha for headache with irritability/dizziness, and Pinelliae Rhizoma plus Bambusae Caulis in Taeniam for vomiting/phlegm | Not assessable | Water-decocted and concentrated to 500 mL; taken warm twice daily after meals; 7 days per course for two courses; wrist-ankle acupuncture also applied | 2 = Limited |
| Fu Hua_2020 | Mahuang Fuzi Xixin Decoction | Not identified in the study | Not stated | Processed Aconite; Asarum; Viticis Fructus; honey-fried Ephedra; Pinellia; Pheretima; Eupolyphaga/Tubiechong; Chuanxiong; Inula flower; Haliotidis Concha (Shijueming); Prunella spike; Astragalus; Pseudostellaria, as listed in the discussion/method-derived text from the uploaded copy | Not assessable | The formula was mixed and decocted; the uploaded retrievable text confirms combined use with flunarizine, but full standardized preparation details are incomplete in the currently retrievable excerpt | 2 = Limited |
| Liu Yan_2022 | Mahuang Fuzi Xixin Decoction | Not identified in the study | The discussion explicitly frames it as a classical prescription | The retrievable uploaded text clearly confirms the core 3-herb formula: Ephedra, Aconite, and Asarum; the current uploaded excerpts do not preserve a clean methods-level full dose line | Not assessable | Not fully extractable from the currently retrievable uploaded text | 2 = Limited |
| Li Dan_2019 | Mahuang Fuzi Xixin Decoction | Not identified in the study | Not stated | Honey-fried Ephedra 10 g; Asarum 6-10 g; Aconite 12-30 g; Angelicae Sinensis Radix 10 g; Scorpio 3 pieces; Chuanxiong Rhizoma 12 g; Carthami Flos 10 g; modifications: Evodiae Fructus 6 g and Ligustici Rhizoma 10 g for vertex pain; Angelicae Dahuricae Radix 10 g for frontal pain; Notopterygii Rhizoma 15 g and Puerariae Lobatae Radix 15 g for occipital pain | Ephedra sinica Stapf, Asarum spp., Aconitum carmichaelii Debeaux, Angelica sinensis (Oliv.) Diels, Buthus martensii Karsch, Ligusticum chuanxiong Hort., Carthamus tinctorius L., Tetradium ruticarpum (A.Juss.) T.G.Hartley, Ligusticum sinense Oliv. / related accepted source, Angelica dahurica (Hoffm.) | 1 dose/day, administered twice daily for 2 weeks; combined with daily Linggui Bafa acupuncture, 1 week per course for 2 courses | 2 = Limited |
| Yang Ganjun_2019 | Fuzi Xixin Decoction | Not identified in the study | Not stated | Not stated | Not assessable | Combined Dabu Yuanjian and Mahuang Fuzi Xixin Decoction administered with western medicine for 3 months; exact composition and preparation details not retrievable from the current uploaded text | 3 = Inadequate |
| Lin Jiandong_2024 | Mahuang Fuzi Xixin Decoction | Not identified in the study | Not stated | Not stated | Not assessable | Combined Mahuang Fuzi Xixin Decoction with flunarizine and balanced acupuncture for 1 month; exact formula composition and preparation details not retrievable from the current uploaded text | 3 = Inadequate |
| Luo Yumei_2021 | Mahuang Fuzi Xixin Decoction | Not identified in the study | Not stated as a formal standard; the article reports use of Mahuang Fuzi Xixin Decoction | Not stated | Not assessable | detailed decoction composition and full preparation method were not recoverable from the currently retrievable uploaded text | 3 = Inadequate |
| Zhao Bohua_2023 | Mahuang Fuzi Xixin Decoction | Not identified in the study | The article reports Mahuang Fuzi Xixin Decoction g deficiency and cold coagulation syndrome | Not extractable | Not assessable |  | 3 = Inadequate |

YXPGT: Yangxue Pinggan Decoction; BXBZTMT: Banxia Baizhu Tianma Decoction; WZYT: Wuzhuyu Decoction; SPT: Sanpian Decoction; XFZYT: Xuefu Zhuyu Decoction; TQHXT: Tongqiao Huoxue decoction; MHFZXXT: Mahuang Fuzi Asarum Decoction

Relevant potential species were inferred only from the medicinal material names reported in the original studies. Where the original report did not provide a sufficiently detailed ingredient list, used non-specific vernacular names only, or did not preserve a reliable methods-level formula description, taxonomic validation was judged as “not assessable”. Mineral and animal-derived medicinal materials were retained as reported and, where appropriate, noted separately from botanical drugs.

Table S4 risk bias of summary

| **study** | **Randomization process** | **Deviations from intended interventions** | **Mising outcome data** | **Measurement of the outcome** | **Selection of the reported result** | **Overall Bias** |
| --- | --- | --- | --- | --- | --- | --- |
| R Liu2020 | Low | Low | Low | Some concerns | Low | Some concerns |
| XY Wu2019 | Low | Low | Some concerns | Low | Low | Low |
| SX Zhang2023 | Low | Some concerns | Low | Some concerns | Low | Some concerns |
| QX Zhang2019 | Low | Low | Some concerns | Low | Some concerns | Some concerns |
| YF Wang2019 | Some concerns | Some concerns | Low | Low | Some concerns | Some concerns |
| DY Li2020 | Some concerns | Low | Low | Low | Low | Low |
| F Yang2022 | Low | Low | Low | Low | Low | Low |
| JH Ou2016 | Low | Some concerns | Low | Low | Some concerns | Some concerns |
| QC Zheng2021 | Low | Low | Low | Low | Low | Low |
| XY Guo2013 | Low | Low | Low | Some concerns | Low | Some concerns |
| SM Chen2003 | Low | Low | Low | Low | Low | Low |
| HW Liu2022 | Low | Low | Low | Low | Low | Low |
| HY Liu2006 | Low | Some concerns | Low | Low | Some concerns | Some concerns |
| HL Zhang2024 | Low | Low | Some concerns | Low | Low | Some concerns |
| ZJ Li2019 | Low | Some concerns | Low | Low | Some concerns | Low |
| HW Liu2018 | Low | Low | Low | Low | Low | Low |
| XH Ge2023 | Low | Some concerns | Low | Low | Low | Low |
| LL Li2025 | Low | Low | Low | Low | Low | Low |
| RJ Li2013 | Low | Low | Low | Low | Low | Low |
| H Li2012 | Low | Low | Some concerns | Low | Low | Some concerns |
| ZK Liang2011 | Low | Low | Low | Some concerns | Low | Some concerns |
| B Shen2016 | Low | Low | Low | Low | Some concerns | Some concerns |
| HB Gu2023 | Low | Some concerns | Low | Low | Low | Some concerns |
| KX Ma2025 | Low | Low | Some concerns | Low | Low | Some concerns |
| HQ Wan2014 | Low | Low | Low | Low | Low | Low |
| YH Liu2015 | Low | Low | Low | Some concerns | Low | Some concerns |
| F Liu2018 | Low | Low | Low | Low | Low | Low |
| HY Shi2023 | Low | Some concerns | Low | Some concerns | Low | Some concerns |
| B Zhang2011 | Low | Low | Low | Some concerns | Low | Some concerns |
| L Cao2015 | Low | Low | Some concerns | Low | Low | Low |
| B li2018 | Low | Some concerns | Low | Some concerns | Low | Some concerns |
| ZS Li2025 | Low | Low | Some concerns | Low | Some concerns | Some concerns |
| P Wang2016 | Some concerns | Some concerns | Low | Low | Some concerns | Some concerns |
| YX Bai2023 | Some concerns | Low | Low | Low | Low | Low |
| LR Cui2017 | Low | Low | Low | Low | Low | Low |
| YS Ma2016 | Low | Some concerns | Low | Low | Some concerns | Some concerns |
| P Wei2015 | Low | Low | Low | Low | Low | Low |
| WX Li2016 | Low | Low | Low | Some concerns | Low | Some concerns |
| TH Li2016 | Low | Low | Low | Low | Low | Low |
| S Ye2023 | Low | Low | Low | Low | Low | Low |
| QL Zhang2021 | Low | Some concerns | Low | Low | Some concerns | Some concerns |
| J Li2016 | Low | Low | Some concerns | Low | Low | Some concerns |
| SC Liang2021 | Low | Some concerns | Low | Low | Some concerns | Low |
| Y Wang2017 | Low | Low | Low | Low | Low | Low |
| CC Su2015 | Low | Some concerns | Low | Low | Low | Low |
| YX Zhao2023 | Low | Low | Low | Low | Low | Low |
| JQ Guo2023 | Low | Low | Low | Low | Low | Low |
| YL Qian2006 | Low | Low | Some concerns | Low | Low | Some concerns |
| SS Chen2014 | Low | Low | Low | Some concerns | Low | Some concerns |
| JN Gao2019 | Low | Low | Low | Low | Some concerns | Some concerns |
| H Fu2020 | Low | Some concerns | Low | Low | Low | Some concerns |
| Y Liu2022 | Low | Low | Some concerns | Low | Low | Some concerns |
| D Li2019 | Low | Low | Low | Low | Low | Low |
| GJ Yang2019 | Low | Low | Low | Some concerns | Low | Some concerns |
| JD Lin2024 | Low | Low | Low | Low | Low | Low |
| YM Luo2021 | Low | Some concerns | Low | Some concerns | Low | Some concerns |
| BH Zhao2023 | Low | Low | Low | Low | Low | Low |

Table S5 GRADE results

| Outcomes | Grade results |
| --- | --- |
| Visual Analog Scale | moderate |
| Headache Frequency | moderate |
| Headache Duration | moderate |
| Efficacy | moderate |
| Adverse Events | moderate |

Table S6 Results of consistency modeling

| Outcomes | Consistency test | Inconsistency test | I2(%) |
| --- | --- | --- | --- |
| visual analogue scale | 128.57 | 128.79 | 14 |
| headache frequency | 164.25 | 164.44 | 4 |
| Duration of headache | 137.40 | 137.39 | 0.5 |
| Efficacy | 134.27 | 134.24 | 0 |
| Adverse events | 109.82 | 109.71 | 39 |

Table S7 visual analogue scale league table

| MD Crl 95% | | | | | | | |
| --- | --- | --- | --- | --- | --- | --- | --- |
| BXBZTMT |  |  |  |  |  |  |  |
| -0.79 (-2.43, 0.84) | Control | -1.52 ( -2.8, -0.25) | -2.86 (-4.2, -1.72) | -1.38 (-2.55, -0.19) |  |  |  |
| 0.73 (-1.35, 2.82) | 1.52 (0.25, 2.8)* | MHFZXXT |  |  |  |  |  |
| 2.06 (0.12, 4.23) * | 2.86 (1.72, 4.2) * | 1.33 (-0.34, 3.21) | SPT |  |  |  |  |
| 0.58 (-1.45, 2.6) | 1.38 (0.19, 2.55) * | -0.15 (-1.89, 1.59) | -1.48 (-3.3, 0.13) | TQHXT |  |  |  |
| 0.67 (-3.16, 4.5) | 1.46 (-2, 4.92) | -0.06 (-3.76, 3.6) | -1.4 (-5.17, 2.18) | 0.08 (-3.58, 3.74) | WZYT |  |  |
| 0.29 (-1.78, 2.36) | 1.09 (-0.19, 2.36) | -0.44 (-2.24, 1.36) | -1.77 (-3.65, -0.11) * | -0.29 (-2.02, 1.46) | -0.38 (-4.05, 3.31) | XFZYT |  |
| 0.19 (-1.98, 2.37) | 0.99 (-0.44, 2.41) | -0.54 (-2.46, 1.37) | -1.87 (-3.88, -0.09) * | -0.39 (-2.24, 1.47) | -0.47 (-4.2, 3.28) | -0.1 (-2.02, 1.8) | YXPGT |

* Means P<0.05

Table S8 headache frequency league table

| MD Crl 95% | | | | | | | |
| --- | --- | --- | --- | --- | --- | --- | --- |
| BXBZTMT |  |  |  |  |  |  |  |
| -1.11 (-2.29, 0.06) | Control |  | -1.54 (-2.56, -0.52) | -1.63 (-2.36, -0.91) |  | -1.69 (-2.28, -1.13) |  |
| -0.02 (-1.69, 1.63) | 1.08 (-0.09, 2.25) | MHFZXXT |  |  |  |  |  |
| 0.43 (-1.13, 1.99) | 1.54 (0.52, 2.56)* | 0.46 (-1.09, 2.01) | SPT |  |  |  |  |
| 0.52 (-0.86, 1.91) | 1.63 (0.91, 2.36) * | 0.55 (-0.82, 1.92) | 0.08 (-1.15, 1.35) | TQHXT |  |  |  |
| -0.39 (-1.96, 1.15) | 0.72 (-0.31, 1.73) | -0.36 (-1.91, 1.18) | -0.82 (-2.27, 0.61) | -0.91 (-2.18, 0.33) | WZYT |  |  |
| 0.58 (-0.71, 1.9) | 1.69 (1.13, 2.28) * | 0.61 (-0.69, 1.92) | 0.15 (-1.01, 1.33) | 0.07 (-0.86, 0.99) | 0.97 (-0.18, 2.15) | XFZYT |  |
| -0.37 (-1.85, 1.12) | 0.74 (-0.18, 1.65) | -0.34 (-1.83, 1.14) | -0.8 (-2.17, 0.56) | -0.89 (-2.07, 0.27) | 0.02 (-1.33, 1.4) | -0.95 (-2.04, 0.12) | YXPGT |

* Means P<0.05

Table S9 duration of headache league table

| MD Crl 95% | | | | | | | |
| --- | --- | --- | --- | --- | --- | --- | --- |
| BXBZTMT |  |  |  |  |  |  |  |
| -3.84 (-7.11, -0.62)* | Control |  |  | -3.03 (-5.27, -0.83) |  | -4.29 (-6.79, -1.79) |  |
| -2.69 (-7.67, 2.24) | 1.15 (-2.61, 4.9) | MHFZXXT |  |  |  |  |  |
| -0.95 (-5.96, 3.96) | 2.89 (-0.88, 6.62) | 1.73 (-3.59, 7.05) | SPT |  |  |  |  |
| -0.81 (-4.75, 3.12) | 3.03 (0.83, 5.27) * | 1.88 (-2.47, 6.24) | 0.15 (-4.19, 4.53) | TQHXT |  |  |  |
| -0.93 (-6.1, 4.17) | 2.91 (-1.1, 6.9) | 1.76 (-3.74, 7.24) | 0.03 (-5.45, 5.51) | -0.12 (-4.72, 4.44) | WZYT |  |  |
| 0.45 (-3.66, 4.53) | 4.29 (1.79, 6.79) * | 3.15 (-1.37, 7.64) | 1.41 (-3.07, 5.95) | 1.26 (-2.1, 4.58) | 1.37 (-3.34, 6.1) | XFZYT |  |
| -2.85 (-7.21, 1.46) | 0.99 (-1.91, 3.88) | -0.16 (-4.91, 4.59) | -1.9 (-6.62, 2.85) | -2.05 (-5.7, 1.58) | -1.93 (-6.85, 3.04) | -3.3 (-7.12, 0.52) | YXPGT |

* Means P<0.05

Table S10 efficacy league table

| OR Crl 95% | | | | | | | |
| --- | --- | --- | --- | --- | --- | --- | --- |
| BXBZTMT |  |  |  |  |  |  |  |
| 5.33 (3.05, 9.68)* | Control | 4.71(2.64, 9.46) | 3.53(2.21, 5.93) | 7.72(4.60, 14.30) | 3.70(1.44, 10.19) | 4.80(3.16, 7.28) | 4.42(1.88, 11.24) |
| 1.08 (0.46, 2.51) | 0.2 (0.11, 0.37) * | MHFZXXT |  |  |  |  |  |
| 1.48 (0.72, 3.15) | 0.28 (0.17, 0.44) * | 1.37 (0.64, 3.03) | SPT |  |  |  |  |
| 0.69 (0.31, 1.52) | 0.13 (0.07, 0.22) | 0.64 (0.28, 1.46) | 0.46 (0.23, 0.94) * | TQHXT |  |  |  |
| 1.5 (0.48, 4.37) | 0.28 (0.1, 0.68) * | 1.39 (0.43, 4.16) | 1.01 (0.34, 2.77) | 2.18 (0.71, 6.21) | WZYT |  |  |
| 1.12 (0.56, 2.3) | 0.21 (0.14, 0.31) * | 1.04 (0.5, 2.21) | 0.76 (0.41, 1.4) | 1.63 (0.84, 3.23) | 0.75 (0.28, 2.2) | XFZYT |  |
| 1.23 (0.43, 3.31) | 0.23 (0.09, 0.51) * | 1.13 (0.38, 3.15) | 0.82 (0.3, 2.1) | 1.78 (0.63, 4.7) | 0.82 (0.23, 2.95) | 1.09 (0.41, 2.68) | YXPGT |

* Means P<0.05

Table S11 adverse events league table

| OR Crl 95% | | | | | | | |
| --- | --- | --- | --- | --- | --- | --- | --- |
| BXBZTMT |  |  |  |  |  |  |  |
| 0.68 (0.25, 1.82) | Control |  |  |  |  |  |  |
| 0.91 (0.24, 3.42) | 1.33 (0.57, 3.21) | MHFZXXT |  |  |  |  |  |
| 2.12 (0.62, 7.18) | 3.1 (1.58, 6.45) * | 2.33 (0.77, 7.17) | SPT |  |  |  |  |
| 1.67 (0.45, 6.34) | 2.46 (1.06, 6.03) * | 1.84 (0.55, 6.39) | 0.79 (0.26, 2.43) | TQHXT |  |  |  |
| 0.76 (0.13, 4.21) | 1.13 (0.26, 4.51) | 0.84 (0.15, 4.33) | 0.36 (0.07, 1.7) | 0.46 (0.08, 2.31) | WZYT |  |  |
| 3.54 (0.93, 14.13) | 5.16 (2.16, 14.02) * | 3.9 (1.12, 14.45) * | 1.67 (0.54, 5.55) | 2.11 (0.6, 7.73) | 4.64 (0.89, 27.19) | XFZYT |  |
| 3.32 (0.75, 17.08) | 4.83 (1.66, 18.23) * | 3.66 (0.91, 17.35) | 1.56 (0.42, 6.87) | 1.98 (0.48, 9.35) | 4.37 (0.74, 30.82) | 0.94 (0.21, 4.52) | YXPGT |


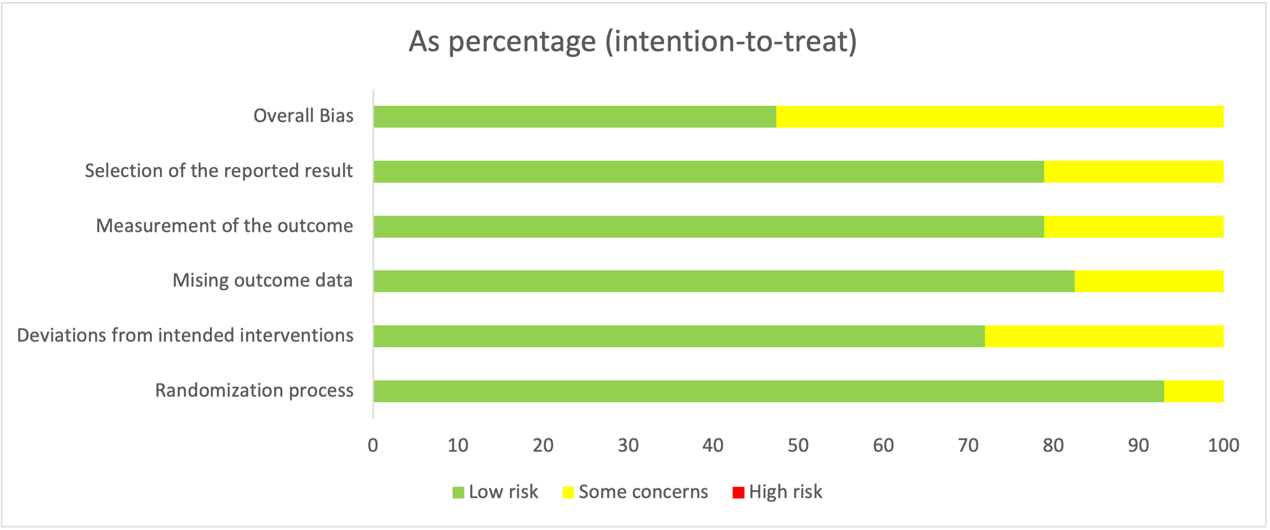


Figure S1 risk bias of graph

Figure S2 Funnel plot of Bayesian network meta-analysis of visual analogue scale

Figure S3 Funnel plot of Bayesian network meta-analysis of headache frequency

Figure S4 Funnel plot of Bayesian network meta-analysis of Duration of headache

Figure S5 Funnel plot of Bayesian network meta-analysis of Efficacy

Figure S6 Funnel plot of Bayesian network meta-analysis of Adverse events
